# Altered ratios of pro‐ and anti‐angiogenic VEGF‐A variants and pericyte expression of DLL4 disrupt vascular maturation in infantile haemangioma

**DOI:** 10.1002/path.4715

**Published:** 2016-05-13

**Authors:** Xi Ye, Yassir Abou‐Rayyah, Joyce Bischoff, Alison Ritchie, Neil J Sebire, Patrick Watts, Amanda J Churchill, David O Bates

**Affiliations:** ^1^ Ophthalmology Unit, School of Clinical SciencesUniversity of BristolUK; ^2^ Cancer Biology Unit, Division of Cancer and Stem Cells, School of MedicineUniversity of NottinghamUK; ^3^ Moorfields Eye HospitalLondonUK; ^4^ Vascular Biology ProgramBoston Children's Hospital, Harvard Medical SchoolMAUSA; ^5^ Histopathology, Great Ormond Street HospitalLondonUK; ^6^ University Hospital of WalesCardiffUK

**Keywords:** haemangioma, vascular endothelial growth factor, angiogenesis, endothelial cell

## Abstract

Infantile haemangioma (IH), the most common neoplasm in infants, is a slowly resolving vascular tumour. Vascular endothelial growth factor A (VEGF‐A), which consists of both the pro‐ and anti‐angiogenic variants, contributes to the pathogenesis of IH. However, the roles of different VEGF‐A variants in IH progression and its spontaneous involution is unknown. Using patient‐derived cells and surgical specimens, we showed that the relative level of VEGF‐A_165_b was increased in the involuting phase of IH and the relative change in VEGF‐A isoforms may be dependent on endothelial differentiation of IH stem cells. VEGFR signalling regulated IH cell functions and VEGF‐A_165_b inhibited cell proliferation and the angiogenic potential of IH endothelial cells in vitro and in vivo. The inhibition of angiogenesis by VEGF‐A_165_b was associated with the extent of VEGF receptor 2 (VEGFR2) activation and degradation and Delta‐like ligand 4 (DLL4) expression. These results indicate that VEGF‐A variants can be regulated by cell differentiation and are involved in IH progression. We also demonstrated that DLL4 expression was not exclusive to the endothelium in IH but was also present in pericytes, where the expression of VEGFR2 is absent, suggesting that pericyte‐derived DLL4 may prevent sprouting during involution, independently of VEGFR2. Angiogenesis in IH therefore appears to be controlled by DLL4 within the endothelium in a VEGF‐A isoform‐dependent manner, and in perivascular cells in a VEGF‐independent manner. The contribution of VEGF‐A isoforms to disease progression also indicates that IH may be associated with altered splicing. © 2016 The Authors. *The Journal of Pathology* published by John Wiley & Sons Ltd on behalf of Pathological Society of Great Britain and Ireland.

## Introduction

Infantile haemangioma (IH) is the most common neoplasm of infancy 1, 2. The natural history of IH is a rapid proliferating phase within the first 12 months of life, followed by spontaneous involution over 7–10 years 2. Haemangioma stem cells (HemSCs) are believed to contribute to the pathogenesis of IH 3 and have been hypothesized to be the parent cells of haemangioma endothelial cells (HemECs), which expand clonally in IH 4. HemSCs proliferation and vasculogenesis are VEGF‐A‐dependent 5 and HemECs from some patients demonstrate a high level of VEGF receptor 2 (VEGFR2) activation compared with normal endothelial cells 6. Thus, VEGF‐A appears to play pivotal roles in the pathogenesis of IH.

The VEGFA gene contains eight exons. Pre‐mRNA alternative splicing controls whether the mRNA contains both exon 8a and 8b or exon 8b alone. The final translated protein product is dependent on this alternative splicing 7, 8. Proximal splicing of exon 8 leads to the translation of the first six amino acids in exon 8a, forming the pro‐angiogenic VEGF‐A_xxx_a family. Distal splicing removes exon 8a from the transcript and results in the translation of the first six amino acids in exon 8b, forming the anti‐angiogenic VEGF‐A_xxx_b. Recently, an additional anti‐angiogenic VEGF‐A variant, VEGF‐Ax, generated by programmed translational readthrough (PTR), has been reported 9. The VEGF‐A_165_a and VEGF‐A_165_b isoforms in the pro‐ and anti‐angiogenic families have similar binding affinities to VEGFR1 and VEGFR2 10, but VEGF‐A_165_b does not bind to neuropilin‐1 (Nrp‐1), the co‐receptor for both VEGF receptors 11. VEGF‐Ax appears to have similar properties to VEGF‐A_165_b, in that it antagonizes VEGF‐A_165_a‐mediated VEGFR2 activation and also does not bind to Nrp‐1 9.

VEGF‐A_165_b, the most investigated isoform of the anti‐angiogenic variants, can inhibit tumour growth and vascularization in xenograft models of colorectal, prostate, melanoma and other cancers 12. The roles of the pro‐ and anti‐angiogenic VEGF‐A variants have not been studied in IH. Due to the close association of VEGF‐A with IH, we set out to determine whether VEGF‐A isoforms contribute to the natural progression of IH and some of the associated mechanisms underlying involution, and to test the hypothesis that anti‐angiogenic VEGF‐A variants could contribute to IH involution.

## Materials and methods

### Human tissues and immunohistochemistry

Formalin‐fixed and paraffin‐embedded human IH specimens were biopsied or excised as part of clinical care (and confirmed diagnostically with histological morphology and characteristic GLUT‐1 expression). Human tissues were collected with ethical approval (No. 07/H0102/45). The stage of IH was divided into proliferating or involuting phases. IH excisions from children up to and including age 1 year were classified as proliferating, whilst excisions from children 3 years or older were classified as involuting. If patient samples were derived from children aged 1–3 years, the histology of the specimens was examined and the stage of IH was confirmed by pathologists.

### Cell culture

IH stem cells (HemSCs), IH endothelial cells (HemECs) and IH pericytes were isolated as described 3, 4, 13 under the human subject protocol approved by the Committee on Clinical Investigation at Boston Children's Hospital. Informed consent was obtained according to the Declaration of Helsinki. HemSCs and HemECs were grown on fibronectin (0.3 µg/cm^2^; Millipore)‐coated plates in endothelial basal medium 2 (EBM‐2; Lonza), with 20% foetal bovine serum (FBS), EBM‐2 SingleQuot without hydrocortisone (Lonza), 2 mm l‐glutamine and 1 × antibiotic–antimycotic (100 U/ml penicillin, 100 µg/ml streptomycin, 250 ng/ml amphotericin B; Gibco). This supplemented growth medium is complete EGM‐2. The HemSCs used were from donors 125, 129 and 133. HemECs were from donors 153, 158 and 159. IH pericytes were cultured on plates coated with 0.5% gelatin and 0.3 µg/cm^2^ fibronectin in Dulbecco's modified Eagle's medium (DMEM) GlutaMax, low‐glucose (Gibco), supplemented with 10% FBS and 2 mm l‐glutamine. Chinese hamster ovary (CHO) cells and normal human dermal fibroblasts (NHDFs) were maintained in RPMI and DMEM media respectively, supplemented with 10% FBS and 2 mm l‐glutamine. The cells were maintained with 5% CO_2_ at 37 °C in a humidified chamber.

### Proliferating cell counts

Cells were plated at 1 × 10^4^ cells/cm^2^ on coverslips in 24‐well plates. The cells were allowed to attach for 24 h and treated for 36 h in complete EGM‐2. The cells were counted, differentiated or subjected to WST1 assay or fixed with 4% paraformaldehyde, permeabilized with PBS‐Triton X (0.1%) and stained with Ki67 antibody (Abcam ab16667) and DAPI. The total number of cells and the number of cells positive for Ki67 in the nucleus were counted in five fields of view.

### Total cell count

Cells were plated at 1 × 10^4^ cells/cm^2^ on coverslips in 24‐well plates. Cells were allowed to attach overnight under normal growth conditions before administration of treatments. One well was removed and its cells fixed in 4% paraformaldehyde and stained with DAPI nuclear stain every 24 h, and cell number was counted microscopically in five random fields of view/coverslip. Cell numbers were normalized to the number of cells attached after 24 h.

### WST‐1 proliferation assay

Cells were plated at 3 × 10^3^ cells/well of a six‐well plate. The cells were left to attach in complete EGM‐2 for 5 h. The medium was replaced with treatment containing EGM‐2 and grown for 36 h. Media and treatments were removed and replaced with EBM‐2 supplemented with 1% FBS and 10% WST‐1 reagent (Roche Applied Bioscience) and incubated under normal culture conditions for 2 h.

### Endothelial differentiation protocol

HemSCs were plated on fibronectin‐coated plates at 2 × 10^4^ cells/cm^2^ in complete EGM‐2 for 12 h. HemSCs were then cultured in endothelial differentiating medium 14 consisting of serum‐free EBM‐2, 1× insulin–transferrin–selenium (Gibco), linoleic acid at 1:5000 dilution, 60 µm ascorbic acid‐2‐phosphate, 1 µm dexamethasone (Sigma‐Aldrich) and VEGF‐B at 10 ng/ml (PeproTech) for 14 days. Fresh endothelial differentiating medium was added every 2 days.

### Adenovirus

Chinese hamster ovary (CHO) cells were infected with recombinant, replication‐deficient adenoviruses expressing GFP or sDLL4 under a CMV promoter (Vector Biolabs) 15 at 100 MOI.

### Protein expression

#### Immunofluorescence

Paraffin‐embedded sections were dewaxed and rehydrated. Antigen retrieval was carried out in citrate buffer (Sigma). Primary antibodies (Pan VEGF‐A, Santa Cruz SC‐152; VEGF‐A_xxx_b, R&D Systems MAB1045; VEGF‐A_XXX_a 16; CD31, Abcam ab28364; DLL4, Abcam ab183532; human CD31, Cell Signaling 3528; and NG2, Abcam ab83178) were incubated overnight at 4 °C. Appropriate AlexaFluor secondary antibodies (Molecular Probes) were incubated for 1 h at room temperature. Five fields of view were recorded from each patient's section. All analyses were carried out masked, ie patient groups were revealed only after the analyses have been completed. Immunofluorescence images were acquired using a Leica DMRB fluorescence microscope fitted with an Olympus DP72 camera, using Olympus CellSens software. For co‐localization, images were taken using a Leica SP8 confocal microscope and LEICA LAS software.

#### Immunoblotting

Cells were plated in six‐well plates and grown until 70–75% confluent. The cells were serum‐starved with EBM‐2 medium for 12–16 h prior to VEGF‐A treatments, then lysed in lysis buffer (200 mm NaCl, 75 mm Trizma base, 1 mm EDTA, 1.5% Triton X, 0.75% Np‐40, 15 mm NaF and 1.5 mm Na_3_VO_4_; Sigma‐Aldrich) on ice for 5 min and subjected to standard immunoblotting (antibody details and blotting conditions are described in Supplementary materials and methods, see supplementary material).

#### VEGF‐A ELISA

VEGF‐A and VEGF‐A_165_b protein levels were assessed using human VEGF‐A and VEGF‐A_165_b DuoSet (R&D Systems), following the manufacturer's instructions.

### Polymerase chain reaction (PCR)

RNA was extracted with TRI‐reagent (Invitrogen); all materials, except primers, were purchased from Promega; 1 µg RNA was DNase‐treated and reverse‐transcribed with M‐MLV reverse transcriptase.

PCR was carried out using a C1000 thermal cycler (Bio‐Rad). Reactions were carried out using the following steps: 95 °C for 10 min (hot start initiation), followed by repeating 95 °C for 30 s, annealing temperature for 30 s and 72 °C for 30 s for up to 35 cycles. A final extension of 72 °C for 10 min was then carried out. Amplified products were run on an ethidium bromide agarose gel and imaged using a Gel doc EZ system (Bio‐Rad).

Quantitative PCR was carried out using lightcycler 480 SYBR Green master (Roche Applied Science) and 0.25 µm gene specific primers, using a Lightcycler 480 real‐time PCR thermal cycler (Roche Applied Science). All reactions were carried out using the protocol specified for PCR for 45 cycles; C t values < 35 were deemed acceptable. Human DLL4 primers were purchased from Qiagen (QT00081004); other primer sequences are listed in Table S1 (see supplementary material). Relative mRNA levels were calculated, normalized to B_2_‐microglobulin (B_2_M) mRNA.

### In vitro co‐culture angiogenesis assay

NHDFs were grown on 19 mm glass coverslips until 70–80% confluent. HemECs (3 × 10^4^) were plated on the NHDF monolayer and allowed to settle for 6 h. The medium was replaced with treatment containing EBM‐2 supplemented with 4% FBS, and the treatment was refreshed every 2 days for 10 days. The cells were fixed with 70% ethanol and stained for VE‐Cadherin (Abcam ab33168) and the nuclei stained with DAPI. Images were acquired from five fields of view at × 10 objective magnification. Area of VE‐Cadherin staining, tubule length and number of branch points were measured and normalized to the area of the field.

### Murine haemangioma model

The murine model was produced as described 5, using 2 × 10^6^ HemSCs and 2 × 10^6^ HemECs resuspended in 200 µl Matrigel (Corning) and injected subcutaneously into the left flank of CD1 nude mice (see supplementary material, Supplementary materials and methods, for details of mouse housing conditions). The mice were also weighed weekly and checked daily by an experienced technician. Implants were measured weekly using Vernier callipers and the volume (V) was calculated using the formula: V = ab ^2^π/6, where a is the longest diameter and b is the longest diameter perpendicular to a. At termination, the explants were removed and fixed with neutral‐buffered formalin and embedded in paraffin or cryopreserved in OCT. Three 5–7 µm non‐consecutive sections positioned 20–30 µm apart were stained with haematoxylin and eosin (H&E), or human‐specific CD31 for microvessel density counting, or with oil red O for fat deposition scoring. The analyses were conducted without knowledge of the treatment groups.

### Statistical analyses

Data are presented as mean ± standard error of the mean (SE). Statistical analyses were carried out using two‐tailed Student's t‐test or ANOVA with Tukey's post hoc test where appropriate, unless stated otherwise; p < 0.05 was considered significant.

## Results

### Increase in the proportion of anti‐angiogenic VEGF‐A isoforms coincides with IH involution

**Figure 1:**
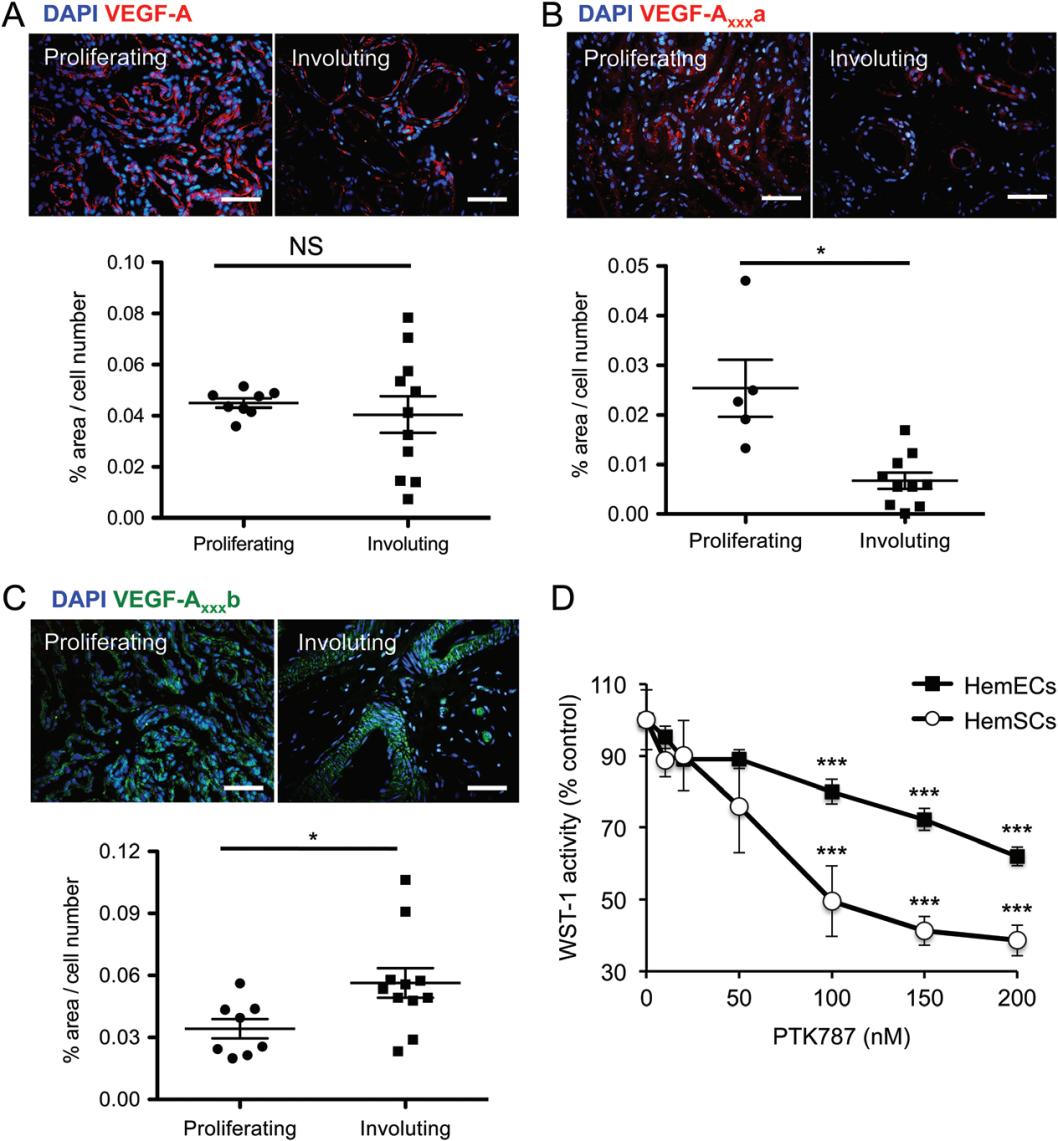
Changes in VEGF‐A isoform levels coincide with IH involution. (A) Tissue from proliferating phase (n = 8 patients) and involuting phase (n = 11 patients) of IH were stained with a total VEGF‐A antibody and showed no differences in total VEGF‐A between the proliferating and involuting phases of IH. (B) The area stained by a VEGF‐A_xxx_a antibody and normalized to cell number showed a significant reduction of VEGF‐A_xxx_a in the involuting (n = 10) phase of IH compared with the proliferating (n = 5) phase (p < 0.05, two‐tailed t‐test). (C) VEGF‐A_xxx_b was increased in the involuting phase compared with the proliferating phase of haemangioma (p < 0.05, two‐tailed t‐test). (D) HemSCs and HemECs were treated with increasing concentrations of PTK787, a VEGFR inhibitor, which significantly inhibited proliferation of HemSCs and HemECs in a concentration‐dependent manner (p < 0.001, one‐way ANOVA, Bonferroni post hoc test; n = 5). Scale bar = 50 µm; *p < 0.05, ***p < 0.001; NS, not significant

Immunofluorescence staining of human IH sections for total VEGF‐A showed no differences in VEGF‐A between the proliferating and the involuting phase of IH (Figure 1A). In contrast, staining with VEGF‐A_xxx_a‐ and VEGF‐A_xxx_b‐specific antibodies (see supplementary material, Figure S1) 8, 16 showed a significant reduction in the level of the pro‐angiogenic VEGF‐A_xxx_a isoform during involution (Figure 1B) and an increase in the level of the anti‐angiogenic VEGF‐A_xxx_b isoforms (Figure 1C). These data suggest that there is an alteration in the ratio of VEGF‐A isoforms, with a relative increase in the proportion of anti‐angiogenic variants during involution.

### VEGFR facilitates IH cell proliferation and changes in VEGF‐A isoforms in IH are associated with differentiation of HemSCs into an endothelial lineage

We questioned whether VEGFR signalling is involved in IH cell functions. We used a commonly used VEGFR inhibitor, PTK787 (see supplementary material, Figure S2), and identified that proliferation of both HemSCs and HemECs was significantly inhibited by PTK787 at concentrations specific to VEGFRs (Figure 1D), supporting the notion that active VEGF‐A signalling is important for IH.

**Figure 2:**
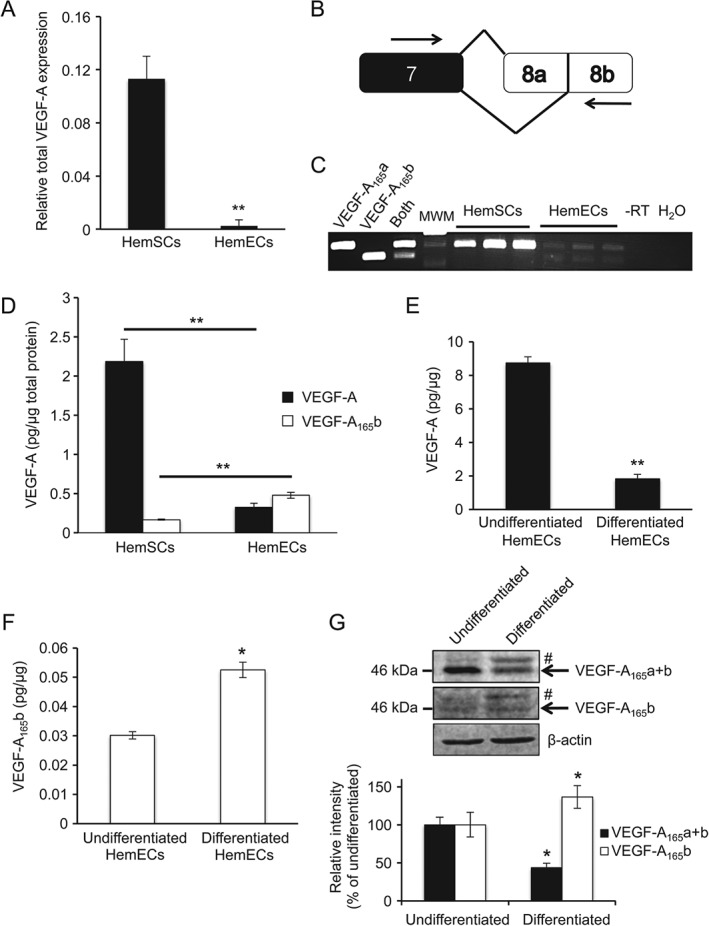
HemECs and endothelial differentiation of HemSCs are associated withVEGF‐A_165_b up‐regulation. (A) Determination of mRNA levels of VEGF‐A in HemECs and HemSCs by RT–qPCR (n = 3 patients). (B) Diagram of RT–PCR primers spanning exons 7 and 8b, thus amplifying both VEGF‐A_165_a and VEGF‐A_165_b. (C) RT–PCR for HemECs and HemSCs and plasmids containing recombinant cDNA for VEGF‐A_165_a and VEGF‐A_165_b, or a mixture of equal amounts of both (n = 3 patients): MWM, molecular weight marker; −RT, no reverse transcriptase. (D) Assessment of total VEGF‐A and VEGF‐A_165_b protein in lysate from HemECs and HemSCs, using sandwich ELISA (n = 3 patients). (E) Measurement of total VEGF‐A in differentiated and undifferentiated HemSCs by ELISA. (F) Measurement of VEGF‐A_165_b in differentiated and undifferentiated HemSCs by ELISA. (G) Immunoblots of protein extracted from undifferentiated or differentiated HemSCs, showing reduced intensity at band size corresponding to both VEGF‐A_165_a and VEGF‐A_165_b, using a total VEGF‐A antibody (arrow) (p < 0.05; n = 3), and increased intensity of band corresponding to VEGF‐A_165_b molecular weight, using the VEGF‐A_165_b antibody (p < 0.05; n = 3) (arrow): an additional band (#) at a higher molecular weight than VEGF‐A_165_a or VEGF‐A_165_b was consistently detected following differentiation of HemSCs; all analysed using two‐tailed t‐test

CD133 was used to isolate multipotent HemSCs from proliferating IH 3, 5, which are lost during IH involution (see supplementary material, Figure S3A), consistent with previous observations 17. HemSCs express higher levels of VEGF‐A compared with NHDFs, HUVECs, HemEPCs 5 and, in this case, also the HemECs (Figure 2A, D). Using primers spanning exons 7 and 8b, which distinguish the pro‐ and anti‐angiogenic VEGF‐A variants (Figure 2B), we found that the relative VEGF‐A_165_b:VEGF‐A_165_a ratio was higher in HemECs when compared with HemSCs, at both the cDNA and protein levels (Figure 2C, D). VEGF‐A_165_b was detected in HemSC protein lysates by ELISA, but not in the mRNA by RT–PCR. This may be explained by the competitive amplification between VEGF‐A_165_a and VEGF‐A_165_b. Using recombinant VEGF‐A cDNAs, we found that when the VEGF‐A_165_a variant concentration was > three‐fold higher than VEGF‐A_165_b, VEGF‐A_165_b detection was completely lost by PCR (see supplementary material, Figure S4). This may also explain the inconsistencies in VEGF‐A_165_b detection using PCR overall, as its amplification is heavily influenced by the level of VEGF‐A_165_a.

Following observations that HemECs produce higher levels of VEGF‐A_165_b compared with HemSCs, we hypothesized that the changes in VEGF‐A isoforms may be differentiation‐dependent. We differentiated HemSCs by treatment for 2 weeks with a different member of the VEGF family, VEGF‐B, a protocol previously shown to induce an endothelial phenotype via VEGFR1 activation 14. We confirmed endothelial differentiation by morphological changes, reduction of the mesenchymal marker CD90 and also acquisition of the endothelial marker VE‐cadherin (see supplementary material, Figure S5). The differentiated cells were then returned to complete EGM‐2 medium for 72 h and we found that the differentiated cells produced a lower level of VEGF‐A using ELISA (Figure 2E; *p <* 0.01) and western blotting (Figure 2G). Furthermore, we also found that the level of VEGF‐A_165_b was significantly increased compared with undifferentiated cells, using ELISA (Figure 2 F; *p <* 0.05) and western blotting (Figure 2G), suggesting that the differentiation process may be at least partially responsible for the differential VEGF‐A isoform expression. An additional band at a slightly higher molecular weight than that of VEGF‐A_165_a or VEGF‐A_165_b was consistently increased following differentiation (Figure 2G). This band was detected using both total VEGF‐A and VEGF‐A_165_b antibodies and may have been the anti‐angiogenic VEGF‐Ax protein recently described 9.

VEGF‐B acts by signalling through VEGFR1. To determine whether acute VEGFR1 activation could induce VEGF‐A_165_b expression directly, and hence up‐regulate VEGF‐A_165_b prior to differentiation, we stimulated HemSCs with VEGF‐B for 36 h (insufficient to result in differentiation). No significant changes in VEGF‐A, VEGF‐A_165_b, SRSF2 expression (see supplementary material, Figure S6A, B) or SRSF6 phosphorylation were observed (see supplementary material, Figure S6C, D). SRSF2 and −6 are other splice factors involved in VEGF‐A splicing 18, 19. Only the phosphorylation of SRSF1, which has been associated with proximal splicing of VEGF‐A in cancer cells and podocytes, was increased by VEGF‐B over this time period (see supplementary material, Figure S6C, D).

### VEGF‐A_165_b inhibits VEGF‐A_165_a mediated HemEC angiogenesis and IH cell proliferation

**Figure 3:**
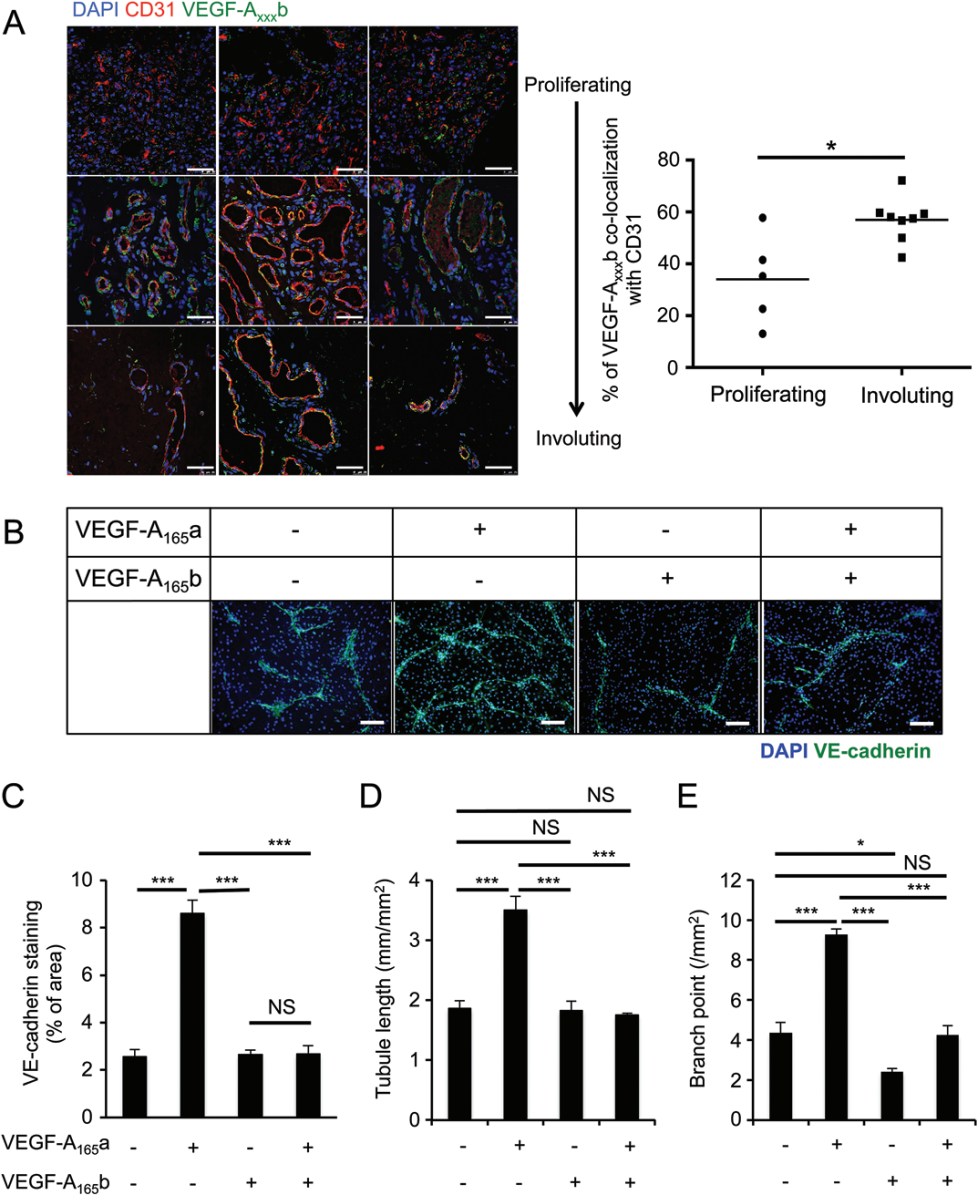
VEGF‐A_165_b regulates sprouting angiogenesis in IH. (A) VEGF‐A_xxx‐_b, CD31 and nuclear (DAPI) staining of human IH sections: histological features of proliferating IH move from early, barely discernable and mostly lumen‐less microvessels (i–iii) to more structured vessels late in the proliferating phase/early involuting phase (iv–vi) and finally microvessels with defined enlarged lumina in the involuting phase (vii–ix); VEGF‐A_xxx_b staining becomes more localized to the microvessels as the endothelia mature and organize into defined luminal structures. (B) HemECs were treated with VEGF‐A_165_a, VEGF‐A_165_b or both on a monolayer of NHDFs, using an in vitro angiogenesis assay: HemECs were stained with VE‐cadherin. (C–E) Quantification of (B): 2.5 nm VEGF‐A_165_a induced tube area (C), length (D) and branching (E), compared with the untreated, 2.5 nm VEGF‐A_165_b or HemECs treated with 2.5 nm VEGF‐A_165_a and VEGF‐A_165_b (n = 3; p < 0.001, one‐way ANOVA). Scale bars = 100 µm

We found VEGF‐A_xxx_b was more localized to the structured endothelia with defined lumen structure, a typical feature of late proliferating and involuting phase (Figure 3Aiv–ix) compared with the disorganized vasculature present in the early stages of the proliferating phase (Figure 3Ai–iii); thus, the relative VEGF‐A isoform ratios and distribution may have a direct functional role in regulating IH angiogenesis/vasculogenesis.

**Figure 4:**
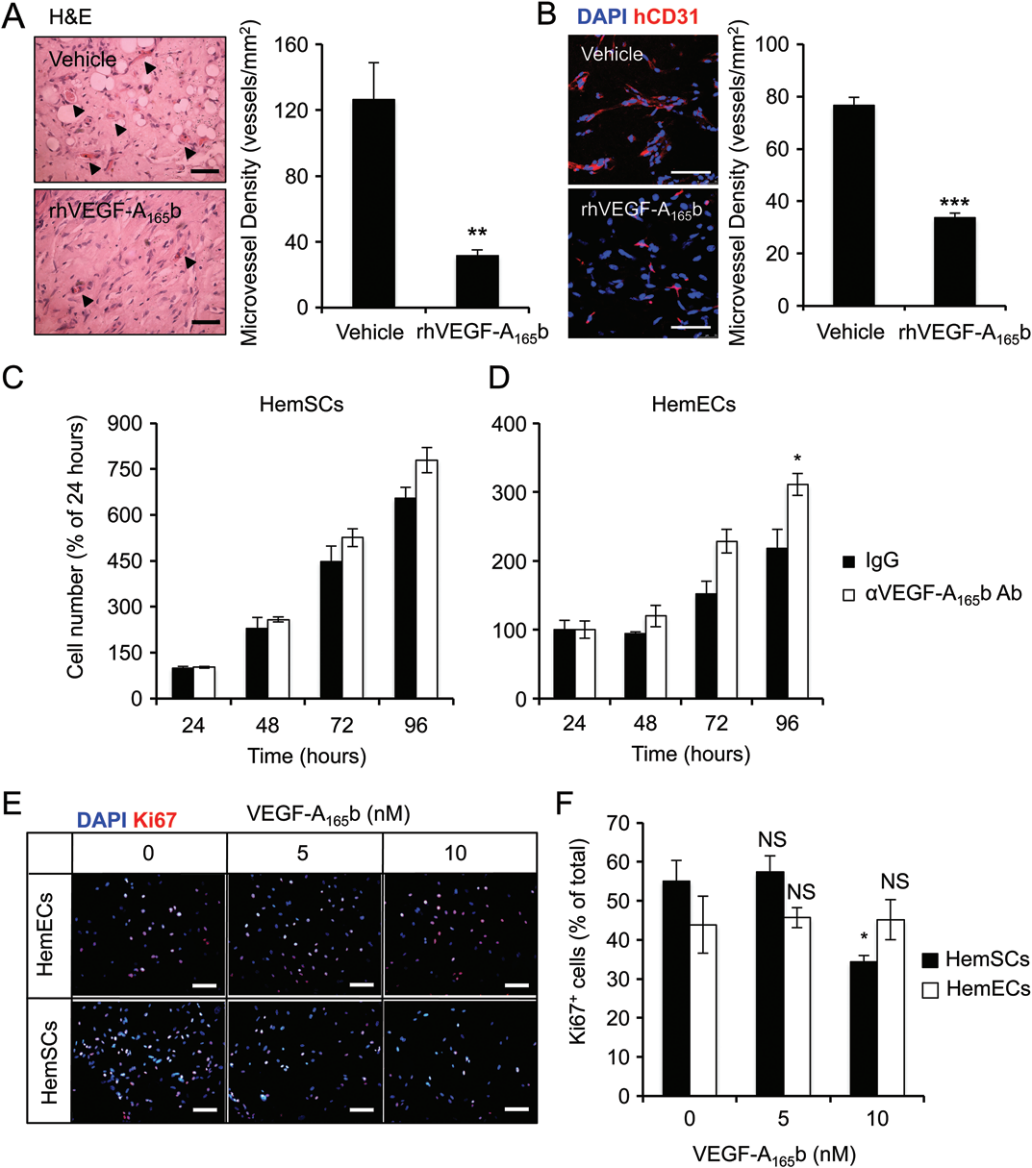
VEGF‐A_165_b inhibits angiogenesis in a murine model of IH and inhibits IH cell proliferation in vitro. (A) HemEC–HemSC–Matrigel implants treated with 15 µg s.c. rhVEGF‐A_165_b (n = 5) or saline (n = 8) twice weekly for 2 weeks. The implants were sectioned, stained with H&E and the blood‐filled lumina were counted (arrow). Implants treated with rhVEGF‐A_165_b showed lower microvessel density than saline‐treated ones (n = 8; p < 0.01). (B) Implants treated with rhVEGF‐A_165_b (n = 6) formed fewer human CD31^+^ microvessels than saline‐treated ones (n = 8; p < 0.001, two‐tailed Student's t‐test). (C, D) HemSCs (C) and HemECs (D) were treated with 1 µg/ml VEGF‐A_165_b neutralizing antibody for 24–96 h in complete EGM‐2: cell numbers at 48, 72 and 96 h were normalized to the number of cells at 24 h (n = 3; p < 0.05, two‐way ANOVA, Bonferroni post hoc test compared with IgG). (E) HemECs were treated in complete EGM‐2 with specified concentrations of rhVEGF‐A_165_b for 48 h; cells were stained for DAPI and Ki67. (F) Quantification of (E): the percentage of proliferating HemECs was unaffected by 5 or 10 nm rhVEGF‐A_165_b; the percentage of proliferating HemSCs was reduced when cells were treated with 10 nm VEGF‐A_165_b compared with untreated ones. Scale bars = 50 µm; *p < 0.05, **p < 0.01, ***p < 0.01; NS, not significant

We used VEGF‐A_165_a as a positive control and induced angiogenesis of HemECs *in vitro*, using a co‐culture angiogenesis assay. VEGF‐A_165_b did not induce HemECs angiogenesis and inhibited VEGF‐A_165_a‐mediated tube formation, extension and branching (Figure 3B–E). A previous study showed that *VEGF‐A* knockdown inhibits HemSCs proliferation and the vascularization of IH implants *in vivo* 5. We also obtained a similar result by treating the HemSCs and cell implants with bevacizumab (see supplementary material, Figure S7A), suggesting the importance of VEGF‐A signalling in the growth and angiogenesis of this IH model. The effect of VEGF‐A_165_b on angiogenesis was also tested using this *in vivo* model, and rhVEGF‐A_165_b reduced the microvessel density in the cell–Matrigel implants (Figure 4A, B). This murine model has been described to acquire adipocytes over time 3, a characteristic feature of human IH pathology. The implants treated with recombinant human VEGF‐A_165_b (rhVEGF‐A_165_b) also showed an increase in fat deposition by oil red O staining (see supplementary material, Figure S7B).

We identified that VEGFR inhibition using PTK787 20 reduced proliferation of both HemECs and HemSCs (Figure 1D). Therefore, we investigated whether endogenous VEGF‐A_165_b has an effect on IH cell proliferation. Inhibition of VEGF‐A_165_b was achieved using a previously characterized antibody 8. VEGF‐A_165_b neutralization increased the growth of HemECs, but not HemSCs, over time compared with non‐specific IgG (Figure 4C, D). This suggests that VEGF‐A_165_b may act in an autocrine manner to limit the proliferation of HemECs, which produce a relatively high proportion of anti‐angiogenic VEGF‐A (Figure 2C, D). HemSCs produce a lower proportion of VEGF‐A_165_b compared with HemECs (Figure 2C, D), and supplementing the growth media with rhVEGF‐A_165_b, significantly inhibited their proliferation (measured using Ki67 as a proliferation marker) (Figure 4E, F). The inhibitory effect of both VEGF‐A_165_b and bevacizumab on HemSCs, but not HemECs, was further confirmed using the WST‐1 proliferation assay (see supplementary material, Figure S8A, B). This suggests that VEGF‐A_165_b from other sources, such as those produced by HemECs, may inhibit HemSCs proliferation in a paracrine manner, displaying an intricate system of proliferation and angiogenic control between the two different cell types.

### Pro‐ and anti‐angiogenic VEGF‐A isoforms differentially regulate VEGFR2 activation, degradation and downstream signalling

**Figure 5:**
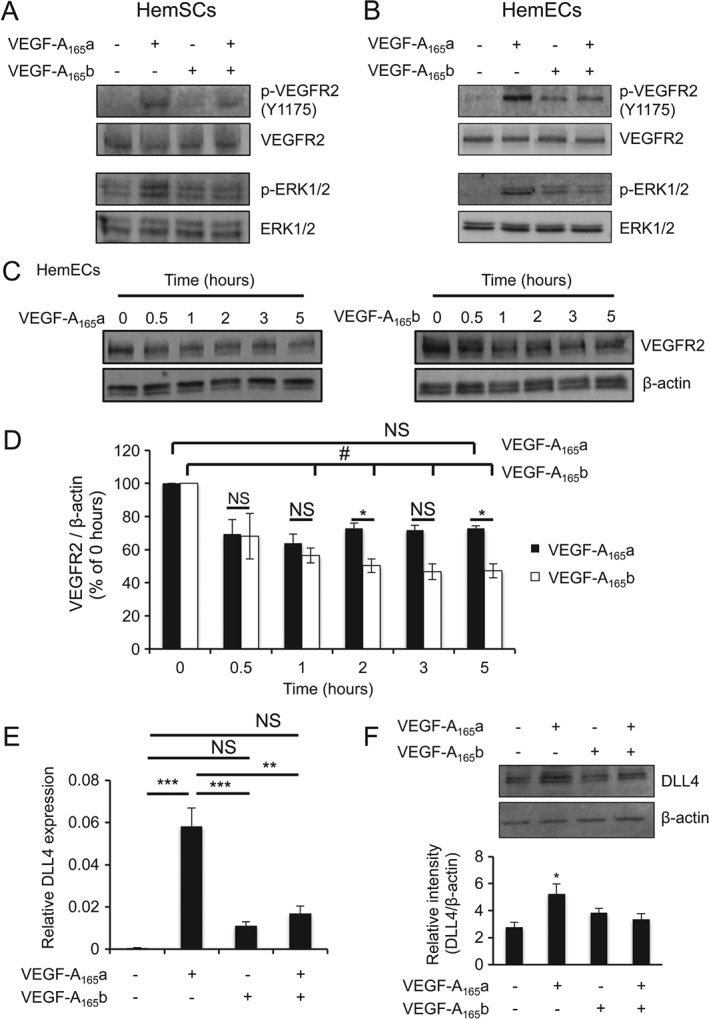
VEGFR2 signalling, degradation and downstream targets are differentially regulated by VEGF‐A isoforms. Cells were serum‐starved prior to treatment with 2.5 nm VEGF‐A_165_a, VEGF‐A_165_b or both. (A, B) VEGF‐A_165_a induced VEGFR2 signalling and downstream activation of ERK1/2 in both HemSCs and HemECs; VEGF‐A_165_b weakly activated VEGFR2 and ERK1/2 in HemSCs and HemECs; VEGF‐A_165_b inhibited VEGF‐A_165_a‐mediated VEGFR2 and ERK1/2 phosphorylation in both HemSCs and HemECs. (C) HemECs were treated with 2.5 nm VEGF‐A_165_a and VEGF‐A_165_b and protein extracted at the time points shown. (D) Quantification of (C): treatment with VEGF‐A_165_b, but not VEGF‐A_165_a, significantly reduced VEGFR2 levels; n = 3; p < 0.05, one‐way ANOVA; *p < 0.05 compared with VEGF‐A_165_b; ^#^ p < 0.05 compared with time 0. (E) HemECs were treated as above and RNA extracted and subjected to RT–qPCR for DLL4 expression; p < 0.001, ANOVA. (F) VEGF‐A_165_a‐induced DLL4 expression compared with untreated (n = 3; p < 0.05, one‐way ANOVA); VEGF‐A_165_b, alone or together with VEGF‐A_165_a, did not induce DLL4 expression at the protein level; *p < 0.05, **p < 0.01, ***p < 0.01; NS, not significant

VEGF‐A mediates the proliferation and angiogenesis of endothelial cells via VEGFR2^21^. Whilst the signalling of VEGFR2 stimulated by VEGF‐A_165_a and VEGF‐A_165_b has been investigated in human microvascular endothelial cells (HMVECs), it has not been conducted in HemECs, an endothelial cell with progenitor‐like gene expression and properties 22, nor in HemSCs. We found that both HemSCs and HemECs respond, in a similar manner to the VEGF‐A isoforms, as normal endothelial cells, wherein VEGF‐A_165_a activates VEGFR2 as well as downstream signalling, which is important for IH cell proliferation, and that this could be blocked with VEGF‐A_165_b (Figure 5A, B; see also supplementary material, Figure S9). Others have demonstrated that the lack of Nrp‐1 signalling promotes VEGFR2 degradation 23, and that VEGF‐A_165_b, which does not bind to Nrp‐1 11, promotes VEGFR2 degradation and/or prevents recycling of the receptor. In HemECs, both VEGF‐A_165_a and VEGF‐A_165_b induced a reduction of VEGFR2 over time. However, the reduction caused by VEGF‐A_165_b stimulation was more prominent than that induced byVEGF‐A_165_a with prolonged stimulations (Figure 5C, D), suggesting that VEGF‐A_165_b may preferentially direct VEGFR2 for degradation, as opposed to recycling.

**Figure 6:**
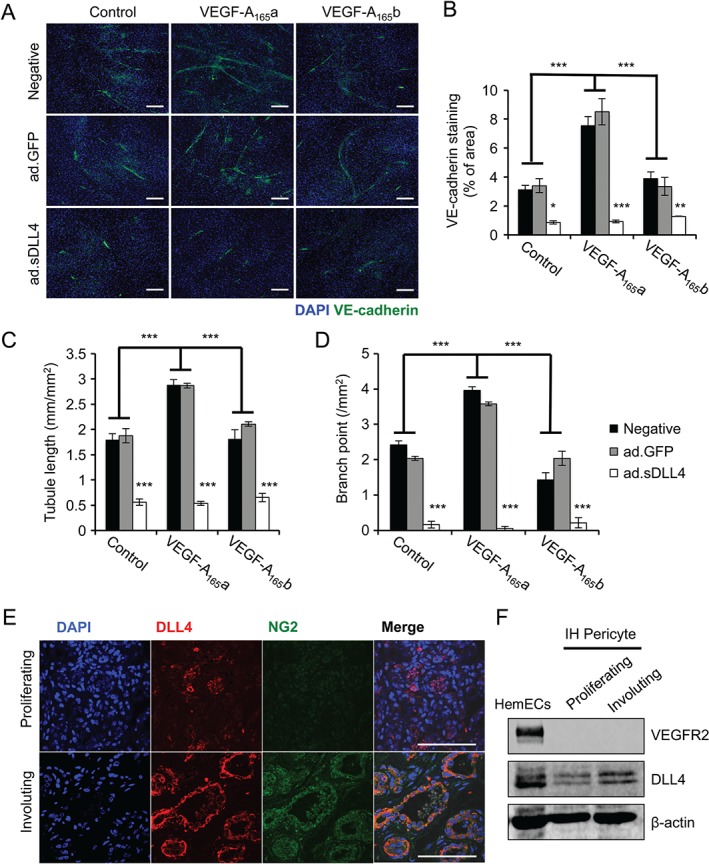
Extra‐endothelial DLL4 signalling inhibits HemECs angiogenesis. (A) HemECs were co‐cultured with normal human dermal fibroblasts and treated with conditioned media from CHO cells, untransduced (negative) or transduced with GFP (Ad.GFP) or sDLL4 adenovirus (ad.DLL4) and VEGF‐A_165_a or VEGF‐A_165_b; the cells were then stained for VE‐cadherin. (B–D) Quantifications of (A): VEGF‐A_165_a, not VEGF‐A_165_b, induced endothelial tube extension, branching and coverage in the assay (n = 3; p < 0.001); conditioned media from CHO cells infected with ad.sDLL4 inhibited the in vitro angiogenic potential of HemECs, irrespective of the VEGF‐A isoforms present (n = 3; p < 0.001, two‐way ANOVA) compared with ad.GFP or untransduced conditioned medium. (E) Sections of IH were stained for NG2 and DLL4: typical stainings of proliferating and involuting IH are shown; relatively low NG2 staining was present in the proliferating phase; in the involuting phase, a relatively high proportion of DLL4 staining co‐localized with NG2‐positive cells in the more organized vasculature. (F) DLL4 protein expression was found in HemECs and IH pericytes, but VEGFR2 protein was undetectable in IH pericytes. Scale bars = 100 µm

The Notch–Delta signalling pathway is one of the most studied pathways in sprouting angiogenesis. Whilst Delta‐like ligand 4 (DLL4) expression can be induced by VEGF‐A_165_a in normal endothelial cells 24 and in HemECs (Figure 5E, F), VEGF‐A_165_b did not induce DLL4 expression to the same extent and was able to ameliorate VEGF‐A_165_a‐mediated DLL4 up‐regulation in HemECs (Figure 5E, F). We tested whether activation of Notch signalling using the soluble portion of DLL4 (sDLL4), previously described 25, can affect the angiogenic potential of HemECs. We overexpressed sDLL4 in CHO cells (see supplementary material, Figure S10), using an adenovirus vector, and used the conditioned medium of the infected cells to treat the HemECs in the *in vitro* angiogenesis assay. sDLL4 blocked the angiogenic tube formation, extension and branching ability of HemECs under all of the conditions tested, including those that were stimulated with VEGF‐A_165_a (Figure 6A–D).

DLL4 up‐regulation normally occurs in the angiogenic tip cells of endothelium 26. However, it is challenging to define angiogenic tip cells in IH sections and we see only a moderate level of co‐localization between DLL4 and microvessels (marked with CD31) in the proliferating phase (see supplementary material, Figure S11i–iii). In the involuting phase of IH, we expected a loss or reduction of DLL4 staining due to termination of excessive angiogenesis. However, DLL4 staining was clearly present in the involuting phase, particularly prominent around microvessels with defined luminal structures, and was found closely associated with the perivascular regions that do not co‐localize with CD31 (see supplementary material, Figure S11iv–vi) and co‐localized with NG2, a pericyte marker, which was less abundant in the proliferating phase (Figure 6E).

### IH pericytes may regulate angiogenesis via DLL4

DLL4 therefore appears to be generated from IH pericytes. These cells have been previously described as having similar levels of VEGF‐A expression 13 and we were able to confirm this (see supplementary material, Figure S12). However, we were unable to detect VEGFR2 protein expression in the IH pericytes (Figure 6 F), although DLL4 was expressed by, and was stronger in, pericytes from involuting lesions than proliferating ones (Figure 6 F). This suggests that, unlike in endothelial cells, DLL4 in IH pericytes is unlikely to be regulated by VEGFR2, but is increased in involuting lesions.

## Discussion

In this study we have shown an increase in the proportion of the anti‐angiogenic VEGF‐A_165_b during the involuting phase of IH. This phenomenon is at least partially governed by the loss of CD133^+^ high VEGF‐A_xxx_a‐expressing HemSCs and its differentiation into an endothelial phenotype, a process that has not previously been shown to regulate VEGF‐A variants. With the recent description of a novel anti‐angiogenic VEGF‐A isoform, VEGF‐A_x_ 9, it is possible that both alternative splicing and post‐translational read‐through could be regulated in this manner.

VEGFR1 mediates HemSCs differentiation into endothelial cells 14 and therefore its activation might also be responsible for the changes in VEGF‐A isoforms. Whilst acute VEGF‐B induced phosphorylation of SRSF1, a splice factor that is often associated with VEGF‐A splicing in podocytes and in cancer cells 18, no changes in total VEGF‐A or VEGF‐A_165_b were observed. However, the relative expression and phosphorylated states of the other members of the SRSF family are likely to have a role in regulating VEGF‐A splicing, even in the event of SRSF1 phosphorylation. The data suggest that acute VEGFR1 activation is insufficient to drive VEGF‐A splicing and that the observations we made may be due to the long‐term cellular reorganization and acquisition of a different cellular phenotype initiated by VEGFR1. The differentiation process affects a large number of genes and further investigations using a proteomics and RNA sequencing approach will be able to provide a greater overview of alternative splicing changes, as well as identification of splice factors that may regulate these events.

We found that VEGF‐A_165_b in IH is closely associated with organized vasculature with defined luminal structures, where VEGF‐A_165_b is capable of inhibiting sprouting angiogenesis and may promote the stabilization of microvessels. VEGF‐A_165_b inhibited VEGF‐A_165_a‐induced angiogenesis of HemECs *in vitro*, an effect that was also observed *in vivo*. Furthermore, endogenously produced VEGF‐A_165_b can inhibit HemECs proliferation, a cell population that produces a relatively high proportion of the anti‐angiogenic VEGF‐A isoforms. It would appear that the endogenously produced VEGF‐A_165_b is insufficient to inhibit growth of HemSCs, which predominantly produce the pro‐angiogenic VEGF‐A variants. However, a high level of VEGF‐A_165_b is inhibitory to HemSCs proliferation. Thus, an intricate autocrine and paracrine effect of VEGF‐A_165_b may exist between the HemSCs and HemECs. We have demonstrated that the maintenance of VEGFR signalling is important for HemSCs and HemECs proliferation. The anti‐proliferative and anti‐angiogenic effects of VEGF‐A_165_b may be attributed to the fact that VEGF‐A_165_b is a weak agonist of VEGFR2 and ameliorates VEGF‐A_165_a‐mediated VEGFR2 activation and downstream signalling, promoting the degradation of VEGFR2 11, 23 as well as lack of DLL4 induction, an important factor in the establishment of endothelial tip cells. Whilst high DLL4 in endothelial tip cells and low DLL4 in the endothelial stalk cells promotes angiogenesis 26, indiscriminate Notch activation in the endothelium is inhibitory to angiogenesis; such is the case with the use of sDLL4. Thus, if VEGF‐A_165_b prevents DLL4 up‐regulation in endothelial cells, then this is likely to prevent tip cell formation and sprouting in involuting cells. However, DLL4 was expressed in involuting lesions, but within the pericytes. This suggests that mural cells may also inhibit sprouting angiogenesis via the Notch–Delta signalling pathway in IH. Unlike endothelial cells, VEGFR2 is not expressed by the IH pericytes and, therefore, it is unlikely that DLL4 is regulated in the same manner between HemECs and IH pericytes. Extra‐endothelial DLL4 in the form of sDLL4 inhibited HemECs angiogenesis in the absence of VEGF‐A_165_a; thus, it is possible that DLL4 from perivascular cells may cooperate with VEGF‐A_165_b to regulate IH involution – VEGF‐A_165_b prevents VEGFR2 signalling in endothelial cells that express it, and pericyte DLL4 inhibits sprouting in cells that are not inhibited by neighbouring DLL4 expression (see supplementary material, Figure S13).

In conclusion, we have identified that IH involution is associated with an increase in the proportion of VEGF‐A_165_b, which is associated with the differentiation of stem cells, a previously undescribed event. VEGF‐A_165_b, the most investigated of the anti‐angiogenic variants, plays a key role in regulating both cell proliferation and disrupting/preventing the up‐regulation of DLL4 and establishment of the tip and stalk endothelial phenotype, demonstrating for the first time the relative proportion of pro‐ and anti‐angiogenic VEGF‐A isoforms as a potential mediator of IH involution. We identified that perivascular cells can express DLL4 in IH, and we hypothesize that it may cooperate with anti‐angiogenic VEGF‐A isoforms to regulate IH involution.

## Author contributions

XY designed and performed the experiments, analysed the results and drafted the manuscript; JB provided the cells and expertise on the *in vivo* model; AR assisted with the *in vivo* experiments; AYR, NJS and PW provided the human IH tissues; and DOB and AJC designed and supervised the work and wrote the manuscript.

## Abbreviations

DLL4, Delta‐like ligand 4; HemEC, haemangioma endothelial cell; HemSC, haemangioma stem cell; IH, infantile haemangioma; Nrp‐1, neuropilin‐1; PTR, programmed translational read‐through; SRSF, serine/arginine‐rich splicing factor; VEGF, vascular endothelial growth factor; VEGFR, vascular endothelial growth factor receptor.

**Supplementary material on the internet**

The following supplementary material may be found in the online version of this article:

**Supplementary materials and methods**

**Figure S1.** VEGF‐A isoform‐specific antibody validation

**Figure S2.** PTK787 inhibits VEGFR2 activation in HemECs

**Figure S3.** Involuting IH is associated with the loss of the CD133^+^ cell population, which differentiates into endothelial cells via VEGFR1 activation

**Figure S4.** Increase in VEGF‐A_165_a reduces VEGF‐A_165_b cDNA detection

**Figure S5.** HemSCs differentiate into endothelial cells via VEGFR1 activation

**Figure S6.** Acute VEGFR1 activation increases SRSF1 phosphorylation but is insufficient to mediate VEGF‐A splicing

**Figure S7.** Bevacizumab inhibits angiogenesis and VEGF‐A_165_b increases adipocyte deposition in IH cell–Matrigel implants

**Figure S8.** VEGF‐A_165_b and bevacizumab inhibit proliferation of HemSCs but not HemECs

**Figure S9.** VEGFR2 and downstream signalling are differentially regulated by pro‐ and anti‐angiogenic VEGF‐A isoforms

**Figure S10.** Soluble DLL4 overexpression in CHO cells

**Figure S11.** Distribution of DLL4 and CD31 in the proliferating and involuting phases of IH

**Figure S12.** Proliferating and Involuting IH pericytes express similar levels of total VEGF‐A

**Figure S13.** Schematic representation of VEGF‐A and DLL4 interactions in IH

**Table S1.** Primer sequences

## Supporting information

Appendix S1. Supplementary methods

Validation of VEGF‐A isoform specific antibodies; 200 ng recombinant human (rh) VEGF‐A_165_a or VEGF‐A_165_b proteins were loaded in the wells. (A) Total VEGF‐A antibody detected both VEGF‐A isoforms. (B) VEGF‐A_xxx_b antibody detected only VEGF‐A_165_b and not VEGF‐A_165_a. (C) VEGF‐A_xxx_b antibody detected only VEGF‐A_165_a and not VEGF‐A_165_b

PTK787 inhibits VEGFR2 activation in HemECs, which were serum‐starved overnight and pretreated with PTK787 at 200 nm for 2 h prior to treatment with 2.5 nm VEGF‐A_165_a for 5 min. VEGF‐A_165_a stimulated VEGFR2 phosphorylation and PTK787 completely blocked VEGFR2 activation at 200 nm in HemECs

Involuting IH is associated with the loss of the CD133^+^ cell population. This differentiates into endothelial cells via VEGFR1 activation. The percentage of cells that showed CD133 staining was reduced in involuting IH (n = 9 patients) compared with the proliferating phase (n = 4 patients; p < 0.01, Mann–Whitney U‐test

Increase in VEGF‐A_165_a cDNA reduces detection of VEGF‐A_165_b cDNA. Plasmids containing VEGF‐A_165_b and VEGF‐A_165_b cDNA were incubated at the concentrations shown and subjected to RT–PCR using primers that detect both isoforms

Differentiation of HemSCs, which were treated with 10 ng/ml VEGF‐B in differentiating medium for 14 days. (A) They lose the mesenchymal spindle‐like morphology and acquire a more epithelial, monolayer‐differentiated phenotype. (B) Protein was extracted from undifferentiated or differentiated cells and subjected to immunoblotting for CD90, a mesenchymal marker, and the endothelial marker VE‐cadherin. Scale bar = 100 µm

Acute VEGFR1 activation increases SRSF1 phosphorylation but is insufficient to mediate VEGF‐A splicing; HemSCs were serum‐starved overnight prior to treatment with 1 nm VEGF‐B. (A) Total VEGF‐A, VEGF‐A_165_b and SRSF2 levels were measured by immunoblotting after 36 h of VEGF‐B treatment. (B) Quantification of (A), normalized to β‐actin (n = 3). (C) HemSCs were treated with VEGF‐B for 12 h; proteins were immunoprecipitated with MAB104, a phosphor‐SR antibody, and immunoblotted for SRSF1 or SRSF6. (D) Quantification of (C); phosphorylated SRs were normalized to total SRSF1 or SRSF6; n = 4; *p < 0.05 compared with control

Bevacizumab inhibits angiogenesis and VEGF‐A165b increase adipocyte deposition of IH cell–Matrigel implants. (A) Cell–Matrigel implants were treated s.c. with saline or 50 µg bevacizumab three times weekly (n = 6), removed and sectioned: blood‐filled lumina were counted; Bevacizumab‐treated mice had lesions that formed significantly fewer microvessels than the vehicle‐treated ones; n = 8; p < 0.01, two‐tailed Student's t‐test. (B) Implants treated with saline (n = 6) or rhVEGF‐A_165_b (n = 6) were stained with oil red O and analysed blind: VEGF‐A_165_b‐treated implants acquired a significantly higher staining score than saline‐treated ones (p < 0.05, Mann–Whitney U‐test). Scale bar = 50 µm

VEGF‐A_165_b and bevacizumab inhibits proliferation of HemSCs but not HemECs. (A) HemSCs and HEmECs were treated with increasing concentrations of VEGF‐A_165_b and proliferation was measured using the WST‐1 assay: VEGF‐A_165_b significantly inhibited proliferation of HemSCs in a concentration‐dependent manner (EC_50_ = 1.5 nm; p < 0.01, one‐way ANOVA); VEGF‐A_165_b did not inhibit the proliferation of HemECs (n = 4). (B) Bevacizumab inhibited proliferation of HemSCs in a concentration‐dependent manner (EC_50_ = 24 nm; p < 0.01, one‐way ANOVA); Bevacizumab did not inhibit the proliferation of HemECs (n = 4)

VEGFR2 and downstream signalling are differentially regulated by pro‐ and anti‐angiogenic VEGF‐A isoforms: quantification of Figure 5A, B. (A) In HemSCs, VEGF‐A_165_a induced VEGFR2 phosphorylation compared with untreated, VEGF‐A_165_b‐treated or co‐treated with VEGF‐A_165_a and VEGF‐A_165_b in combination (p < 0.01). (B) In HemSCs, VEGF‐A_165_a induced ERK1/2 phosphorylation compared with untreated (p < 0.01), VEGF‐A_165_b‐treated or co‐treated with VEGF‐A_165_a and VEGF‐A_165_b in combination (p < 0.05). (C) In HemECs, VEGF‐A_165_a induced VEGFR2 phosphorylation compared with untreated (p < 0.001), VEGF‐A_165_b‐treated or co‐treated with VEGF‐A_165_a and VEGF‐A_165_b in combination (p < 0.01). (D) In HemECs, VEGF‐A_165_a induced ERK1/2 phosphorylation compared with untreated (p < 0.01), VEGF‐A_165_b‐treated or co‐treated with VEGF‐A_165_a and VEGF‐A_165_b in combination (p < 0.05). VEGF‐A_165_b alone or in combination with VEGF‐A_165_a did not elicit significant changes in VEGFR2 or ERK1/2 phosphorylation compared with untreated control (n = 3; one‐way ANOVA)

Soluble DLL4 overexpression in CHO cells. CHO cells were infected with adenovirus for GFP (ad.GFP) or the soluble portion of DLL4 (ad.sDLL4) at 100 MOI. Protein was extracted 3 days post‐infection. Soluble DLL4 was overexpressed in the CHO cells

Distribution of DLL4 and CD31 in the proliferating and involuting phases of IH. Sections of IH were stained for CD31 and DLL4. Typical staining of proliferating and involuting IH are shown. Relatively low DLL4 staining was present in the proliferating phase (i–iii). In the involuting phase, DLL4 staining was prominent in the perivascular regions surrounding the organized microvessels

Proliferating‐ and involuting‐phase IH pericytes express similar levels of total VEGF‐A. IH pericytes from proliferating and involuting phase express similar levels of total VEGF‐A. VEGF‐A_165_b was undetectable in these cells using ELISA

Schematic representation of VEGF‐A and DLL4 interactions in IH. (A) High VEGF‐A_165_a activates VEGFR2 to mediate up‐regulation of DLL4 and subsequent establishment of the endothelial tip cells (EC tip cell); DLL4 from tip cells activates NOTCH in neighbouring cells to down‐regulate DLL4 and maintain a stalk phenotype (EC stalk cell); the tip cells guide the sprouting events in angiogenesis. (B) In the involuting phase, high VEGF‐A_165_b competes against VEGF‐A_165_a to prevent DLL4 up‐regulation and induction of tip cells does not occur; instead, DLL4 from neighbouring pericytes activates NOTCH in the endothelial cells to prevent up‐regulation of DLL4

Primer sequences
