# Supplementary material for: Altered ratios of pro‐ and anti‐angiogenic VEGF‐A variants and pericyte expression of DLL4 disrupt vascular maturation in infantile haemangioma
Source: J Pathol. 2016 May 13;239(2):139–51. doi: 10.1002/path.4715 (PMC4869683; doi:10.1002/path.4715)
Supplement: Supplementary file 1 — Appendix S1. Supplementary methods [file PATH-239-139-s001.docx]

+A: **Supplementary materials and methods**

+B: Murine haemangioma model

Immunodeficient male CD-1 Nu/Nu mice (Charles River), 6 weeks old, were maintained in IVCs (Tecniplast) within a barrier unit illuminated by fluorescent lights set, to give a 12 h light/dark cycle (on 07.00, off 19.00) as recommended in the UK Home Office Animals (Scientific Procedures) Act (1986). The room was air-conditioned by a system designed to maintain an air temperature range of 21 ± 2ºC and a humidity of 55% ± 10%. The mice were housed in social groups during the procedure, with irradiated bedding and provided with autoclaved nesting materials and environmental enrichment. Sterile irradiated 2919 rodent diet (Harlan Teklad) and autoclaved water was offered *ad libitum*. This study was conducted under UK Home Office Licence No. PPL 40/3559, 19b (3). NCRI guidelines for the welfare and use of animals in cancer research, LASA Good Practice guidelines and FELASA Working Group on Pain and Distress guidelines were also followed.

+B: Immunoblotting details

Samples were heated to 95°C in ×1 sample buffer (5.8 mm Tris–HCl, pH 6.8, 1.7% SDS, 5% glycerol, 0.0017% bromophenol blue). Proteins were run in 4–20% miniProtean TGX precast gel (Bio-Rad) at 100 V. VEGFR2 and p-VEGFR2 were transferred onto a PVDF membrane at 0.15 A for 20–22 h at 4°C. All other proteins were transferred at 90 V for 2 h at 4°C. The membranes were blocked with 3% BSA in TBS-T (20 mm Trizma base, 137 mm NaCl, pH 7.6, 0.1% Tween-20) and probed with antibodies against VEGFR2, p-VEGFR2 (Y1175), ERK1/2 and p-ERK1/2 (Cell Signalling; 2479, 2478, 9102 and 4376), total VEGF-A (Santa Cruz; SC152), VEGF-A165b (R&D Systems; MAB3045), Actin (Santa Cruz; SC1616) and DLL4 (Abcam; ab183532) overnight at 4°C. Signals were detected using near-infrared antibodies (Li-Cor) at 1:7000 dilution for 2 h at room temperature, followed by imaging in the Odessy Fc dual-mode imaging system (Li-Cor).
