# Supplementary figures and images for: Altered ratios of pro‐ and anti‐angiogenic VEGF‐A variants and pericyte expression of DLL4 disrupt vascular maturation in infantile haemangioma
Source: J Pathol. 2016 May 13;239(2):139–51. doi: 10.1002/path.4715 (PMC4869683; doi:10.1002/path.4715)

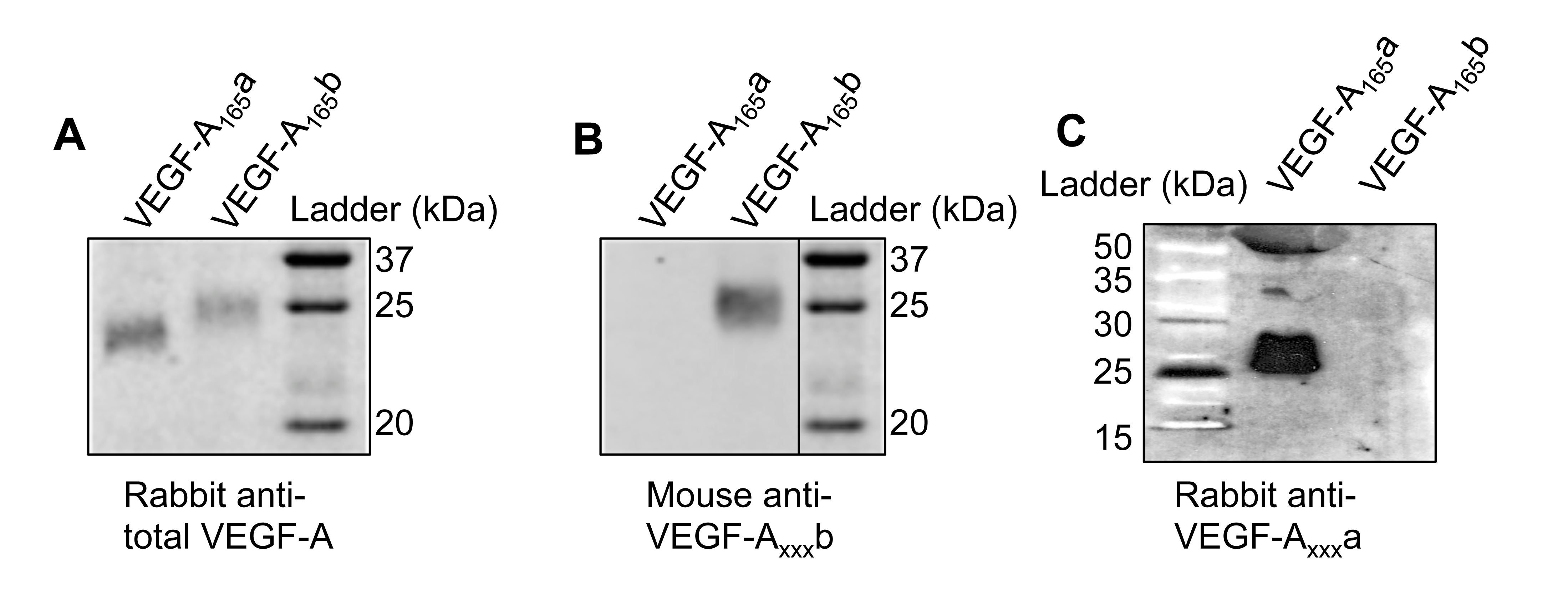

Supplement: Supplementary file 2 — Validation of VEGF‐A isoform specific antibodies; 200 ng recombinant human (rh) VEGF‐A165a or VEGF‐A165b proteins were loaded in the wells. (A) Total VEGF‐A antibody detected both VEGF‐A isoforms. (B) VEGF‐Axxxb antibody detected only VEGF‐A165b and not VEGF‐A165a. (C) VEGF‐Axxxb antibody detected only VEGF‐A165a and not VEGF‐A165b [file PATH-239-139-s002.tif]

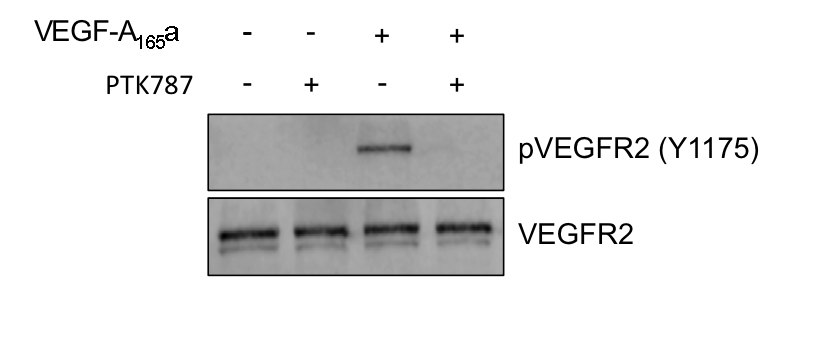

Supplement: Supplementary file 3 — PTK787 inhibits VEGFR2 activation in HemECs, which were serum‐starved overnight and pretreated with PTK787 at 200 nm for 2 h prior to treatment with 2.5 nm VEGF‐A165a for 5 min. VEGF‐A165a stimulated VEGFR2 phosphorylation and PTK787 completely blocked VEGFR2 activation at 200 nm in HemECs [file PATH-239-139-s003.tif]

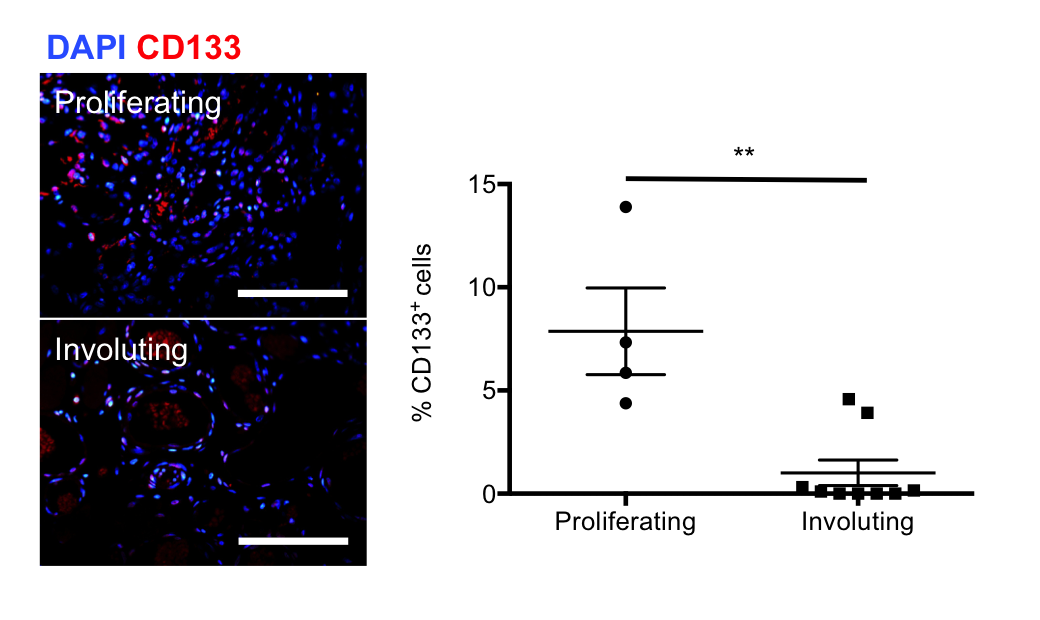

Supplement: Supplementary file 4 — Involuting IH is associated with the loss of the CD133+ cell population. This differentiates into endothelial cells via VEGFR1 activation. The percentage of cells that showed CD133 staining was reduced in involuting IH (n = 9 patients) compared with the proliferating phase (n = 4 patients; p < 0.01, Mann–Whitney U‐test [file PATH-239-139-s004.tif]

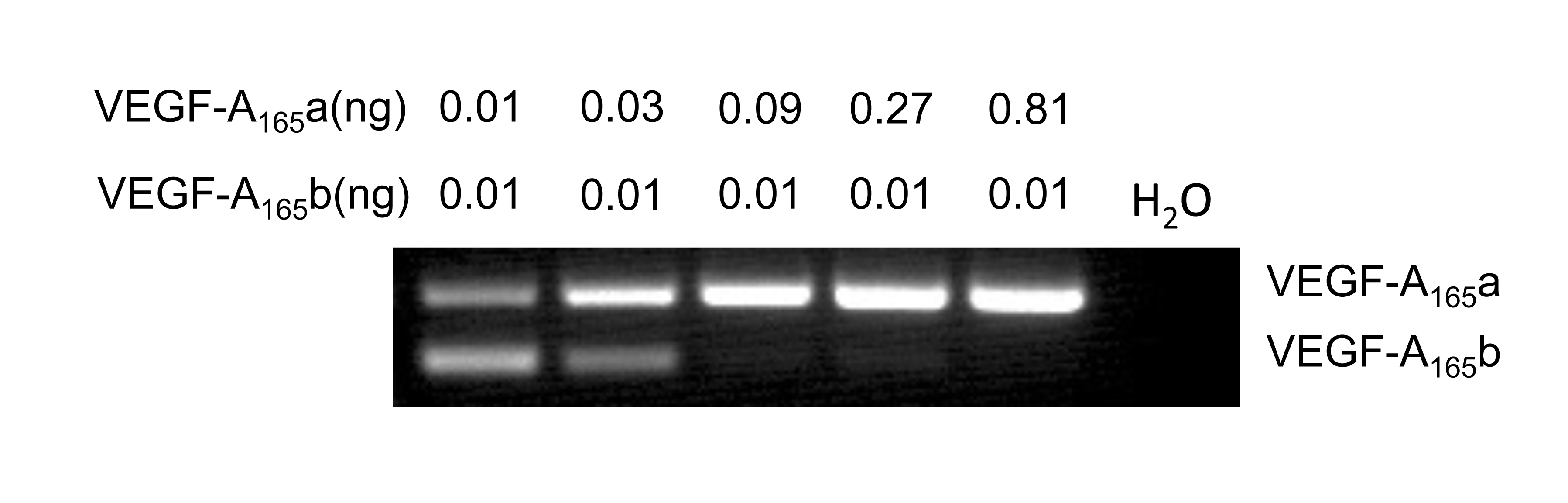

Supplement: Supplementary file 5 — Increase in VEGF‐A165a cDNA reduces detection of VEGF‐A165b cDNA. Plasmids containing VEGF‐A165b and VEGF‐A165b cDNA were incubated at the concentrations shown and subjected to RT–PCR using primers that detect both isoforms [file PATH-239-139-s005.tif]

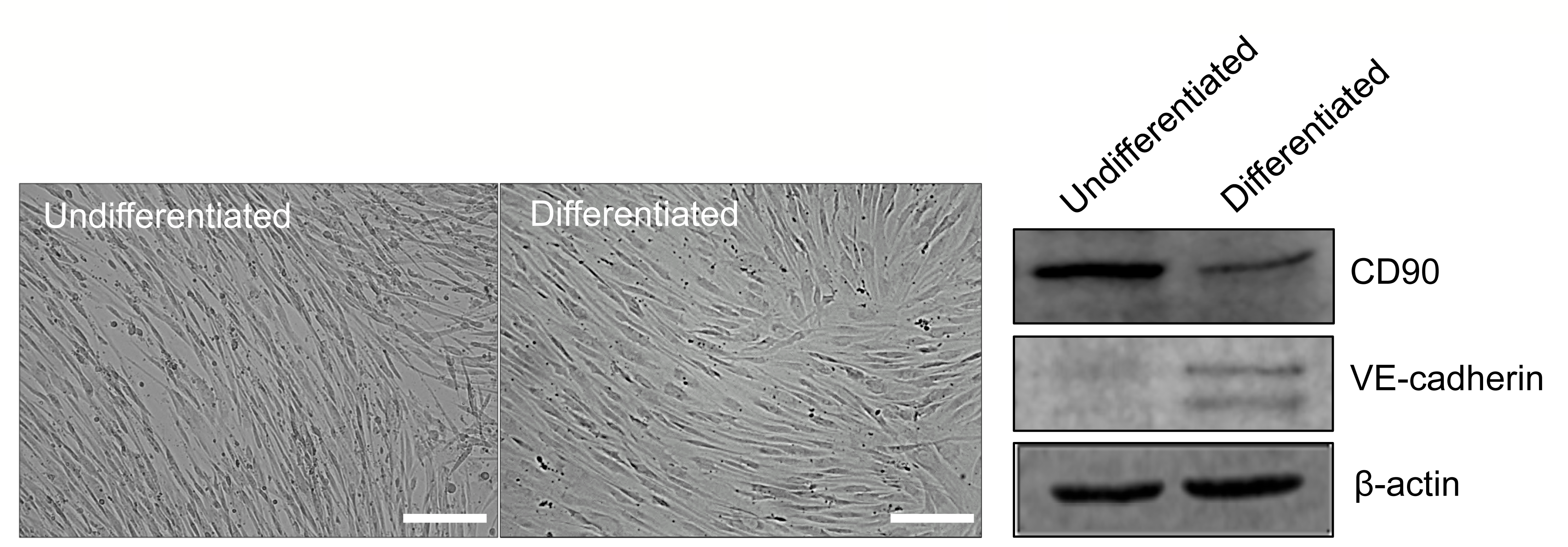

Supplement: Supplementary file 6 — Differentiation of HemSCs, which were treated with 10 ng/ml VEGF‐B in differentiating medium for 14 days. (A) They lose the mesenchymal spindle‐like morphology and acquire a more epithelial, monolayer‐differentiated phenotype. (B) Protein was extracted from undifferentiated or differentiated cells and subjected to immunoblotting for CD90, a mesenchymal marker, and the endothelial marker VE‐cadherin. Scale bar = 100 µm [file PATH-239-139-s006.tif]

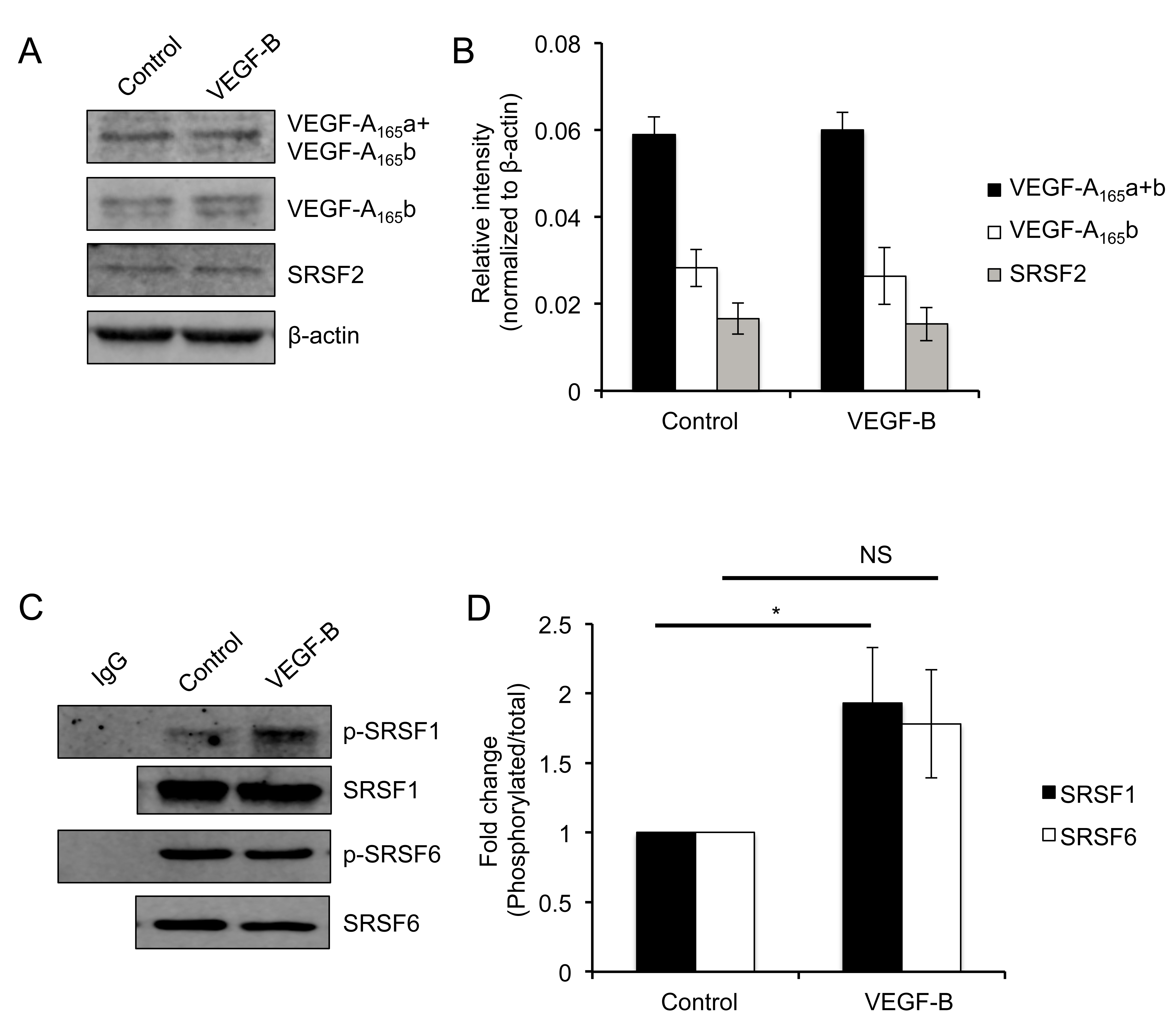

Supplement: Supplementary file 7 — Acute VEGFR1 activation increases SRSF1 phosphorylation but is insufficient to mediate VEGF‐A splicing; HemSCs were serum‐starved overnight prior to treatment with 1 nm VEGF‐B. (A) Total VEGF‐A, VEGF‐A165b and SRSF2 levels were measured by immunoblotting after 36 h of VEGF‐B treatment. (B) Quantification of (A), normalized to β‐actin (n = 3). (C) HemSCs were treated with VEGF‐B for 12 h; proteins were immunoprecipitated with MAB104, a phosphor‐SR antibody, and immunoblotted for SRSF1 or SRSF6. (D) Quantification of (C); phosphorylated SRs were normalized to total SRSF1 or SRSF6; n = 4; *p < 0.05 compared with control [file PATH-239-139-s007.tif]

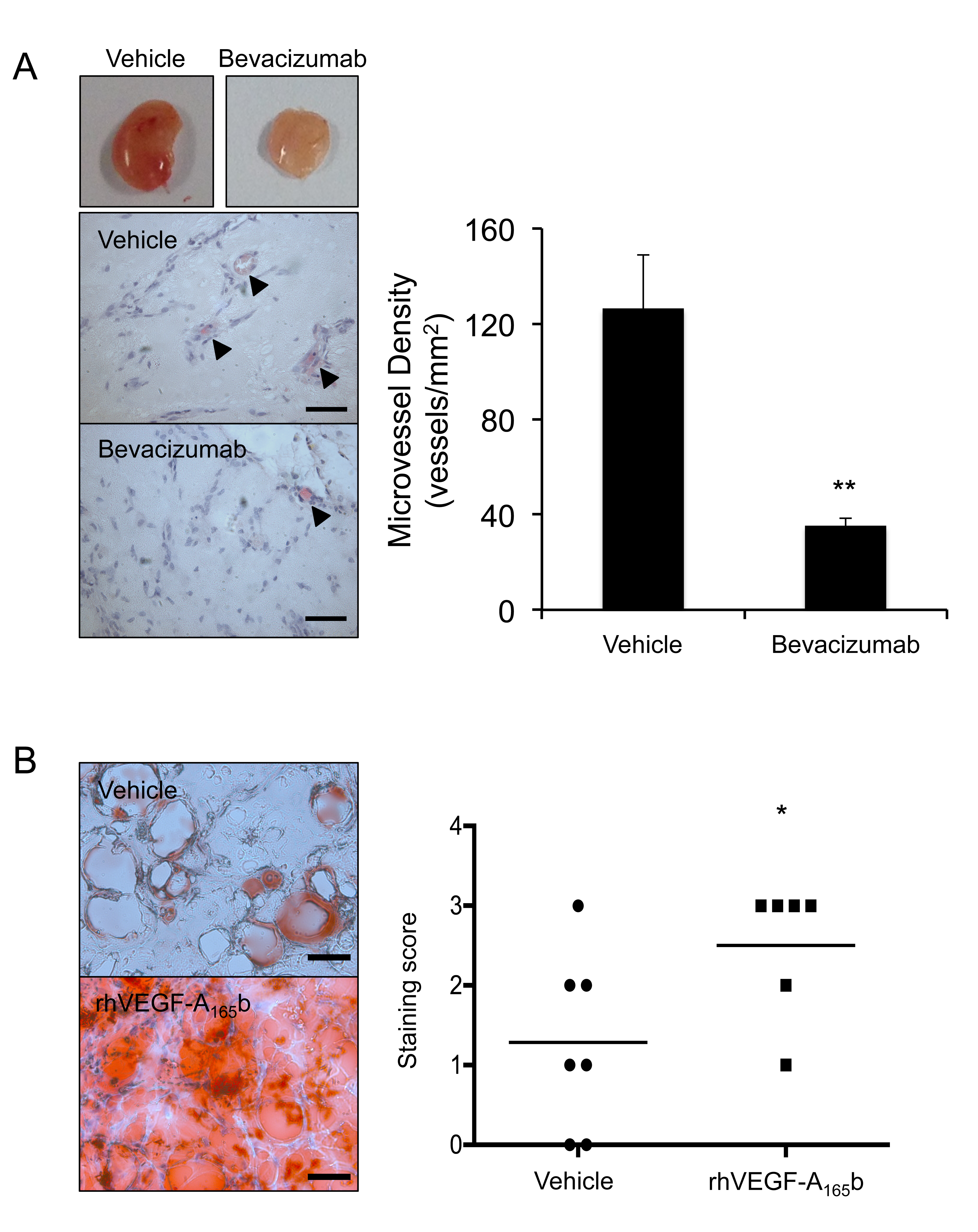

Supplement: Supplementary file 8 — Bevacizumab inhibits angiogenesis and VEGF‐A165b increase adipocyte deposition of IH cell–Matrigel implants. (A) Cell–Matrigel implants were treated s.c. with saline or 50 µg bevacizumab three times weekly (n = 6), removed and sectioned: blood‐filled lumina were counted; Bevacizumab‐treated mice had lesions that formed significantly fewer microvessels than the vehicle‐treated ones; n = 8; p < 0.01, two‐tailed Student's t‐test. (B) Implants treated with saline (n = 6) or rhVEGF‐A165b (n = 6) were stained with oil red O and analysed blind: VEGF‐A165b‐treated implants acquired a significantly higher staining score than saline‐treated ones (p < 0.05, Mann–Whitney U‐test). Scale bar = 50 µm [file PATH-239-139-s008.tif]

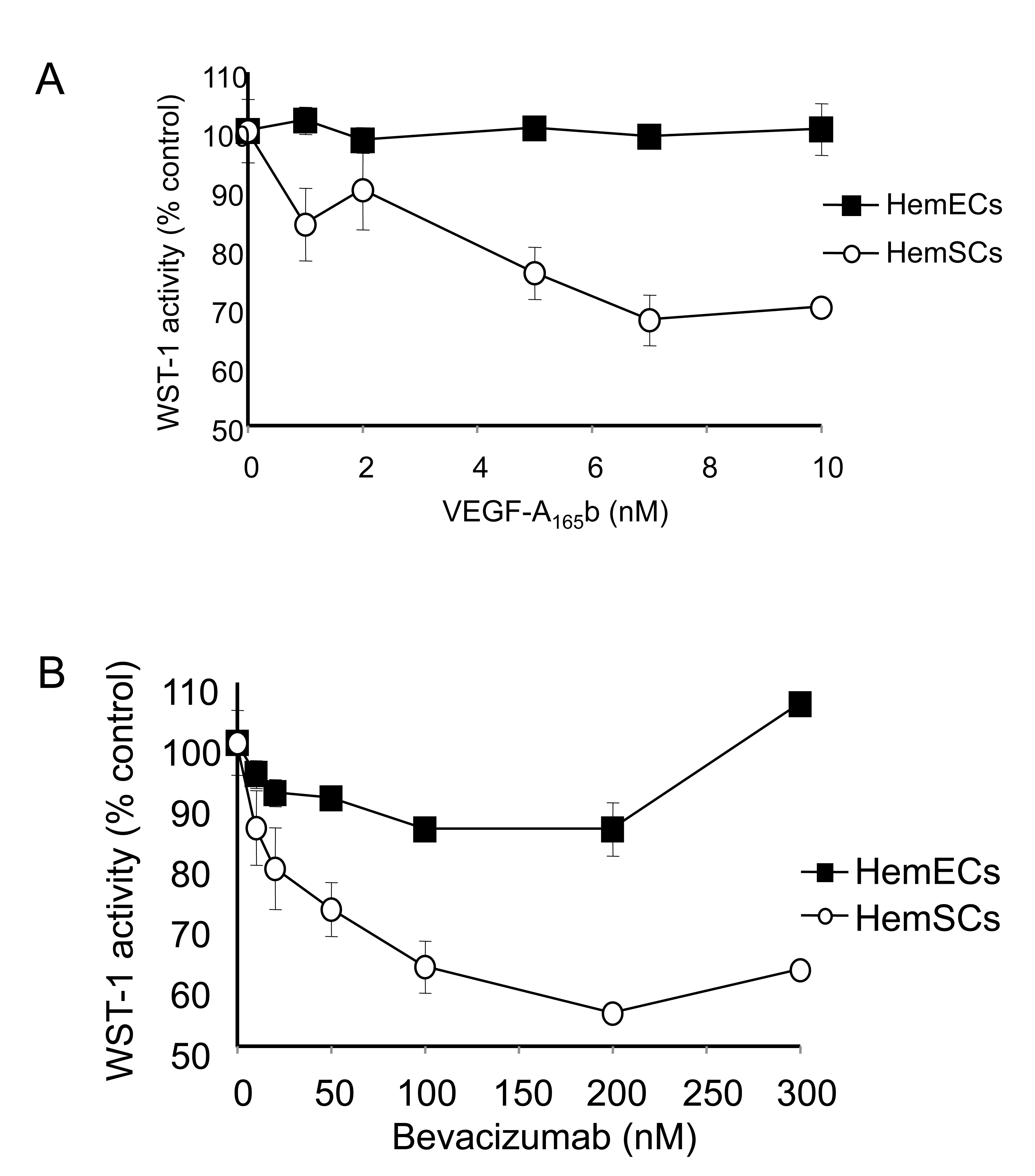

Supplement: Supplementary file 9 — VEGF‐A165b and bevacizumab inhibits proliferation of HemSCs but not HemECs. (A) HemSCs and HEmECs were treated with increasing concentrations of VEGF‐A165b and proliferation was measured using the WST‐1 assay: VEGF‐A165b significantly inhibited proliferation of HemSCs in a concentration‐dependent manner (EC50 = 1.5 nm; p < 0.01, one‐way ANOVA); VEGF‐A165b did not inhibit the proliferation of HemECs (n = 4). (B) Bevacizumab inhibited proliferation of HemSCs in a concentration‐dependent manner (EC50 = 24 nm; p < 0.01, one‐way ANOVA); Bevacizumab did not inhibit the proliferation of HemECs (n = 4) [file PATH-239-139-s009.tif]

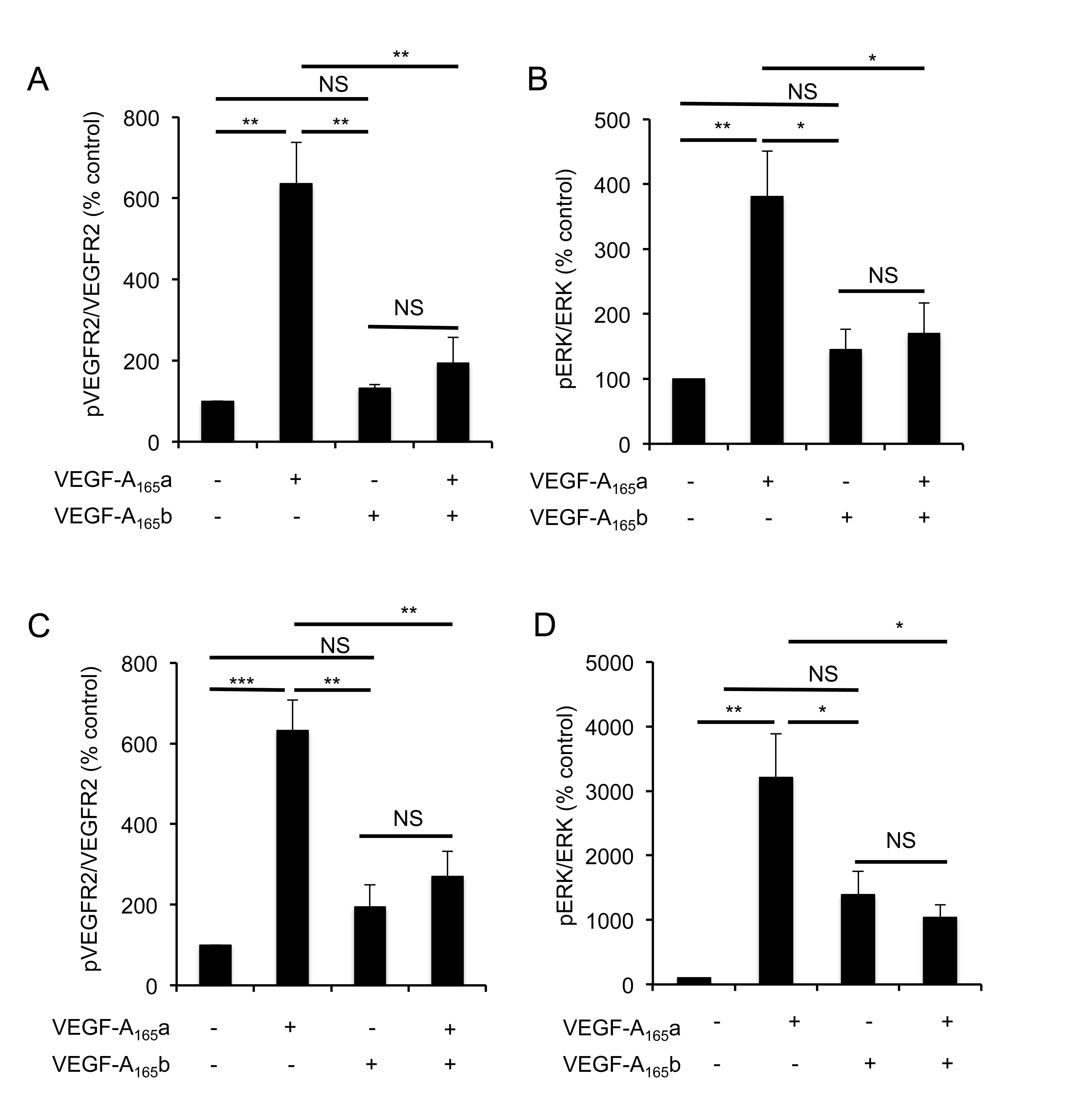

Supplement: Supplementary file 10 — VEGFR2 and downstream signalling are differentially regulated by pro‐ and anti‐angiogenic VEGF‐A isoforms: quantification of Figure 5A, B. (A) In HemSCs, VEGF‐A165a induced VEGFR2 phosphorylation compared with untreated, VEGF‐A165b‐treated or co‐treated with VEGF‐A165a and VEGF‐A165b in combination (p < 0.01). (B) In HemSCs, VEGF‐A165a induced ERK1/2 phosphorylation compared with untreated (p < 0.01), VEGF‐A165b‐treated or co‐treated with VEGF‐A165a and VEGF‐A165b in combination (p < 0.05). (C) In HemECs, VEGF‐A165a induced VEGFR2 phosphorylation compared with untreated (p < 0.001), VEGF‐A165b‐treated or co‐treated with VEGF‐A165a and VEGF‐A165b in combination (p < 0.01). (D) In HemECs, VEGF‐A165a induced ERK1/2 phosphorylation compared with untreated (p < 0.01), VEGF‐A165b‐treated or co‐treated with VEGF‐A165a and VEGF‐A165b in combination (p < 0.05). VEGF‐A165b alone or in combination with VEGF‐A165a did not elicit significant changes in VEGFR2 or ERK1/2 phosphorylation compared with untreated control (n = 3; one‐way ANOVA) [file PATH-239-139-s010.tif]

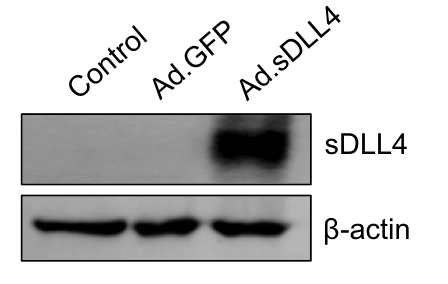

Supplement: Supplementary file 11 — Soluble DLL4 overexpression in CHO cells. CHO cells were infected with adenovirus for GFP (ad.GFP) or the soluble portion of DLL4 (ad.sDLL4) at 100 MOI. Protein was extracted 3 days post‐infection. Soluble DLL4 was overexpressed in the CHO cells [file PATH-239-139-s011.tif]

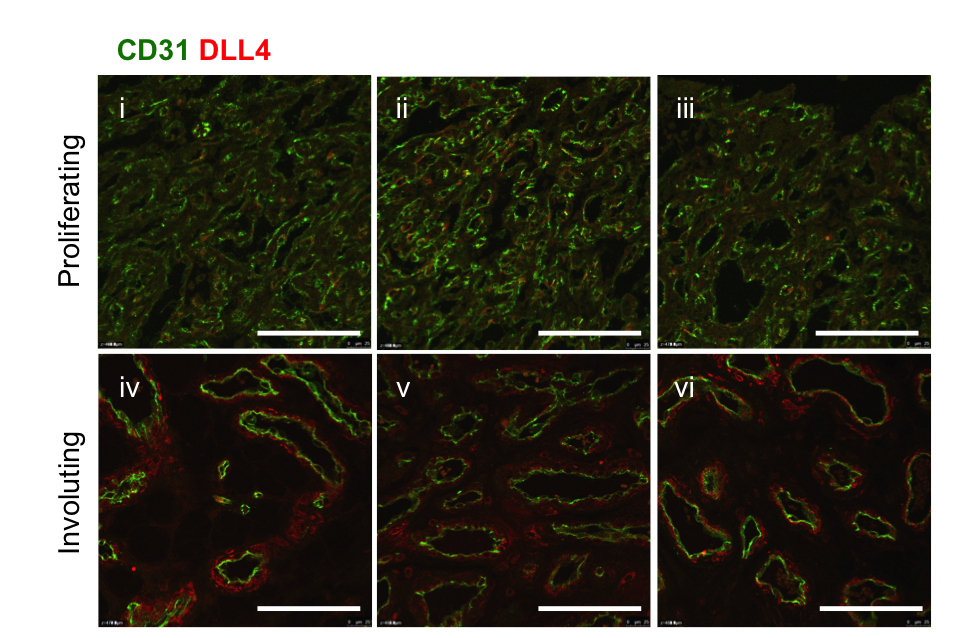

Supplement: Supplementary file 12 — Distribution of DLL4 and CD31 in the proliferating and involuting phases of IH. Sections of IH were stained for CD31 and DLL4. Typical staining of proliferating and involuting IH are shown. Relatively low DLL4 staining was present in the proliferating phase (i–iii). In the involuting phase, DLL4 staining was prominent in the perivascular regions surrounding the organized microvessels [file PATH-239-139-s012.tif]

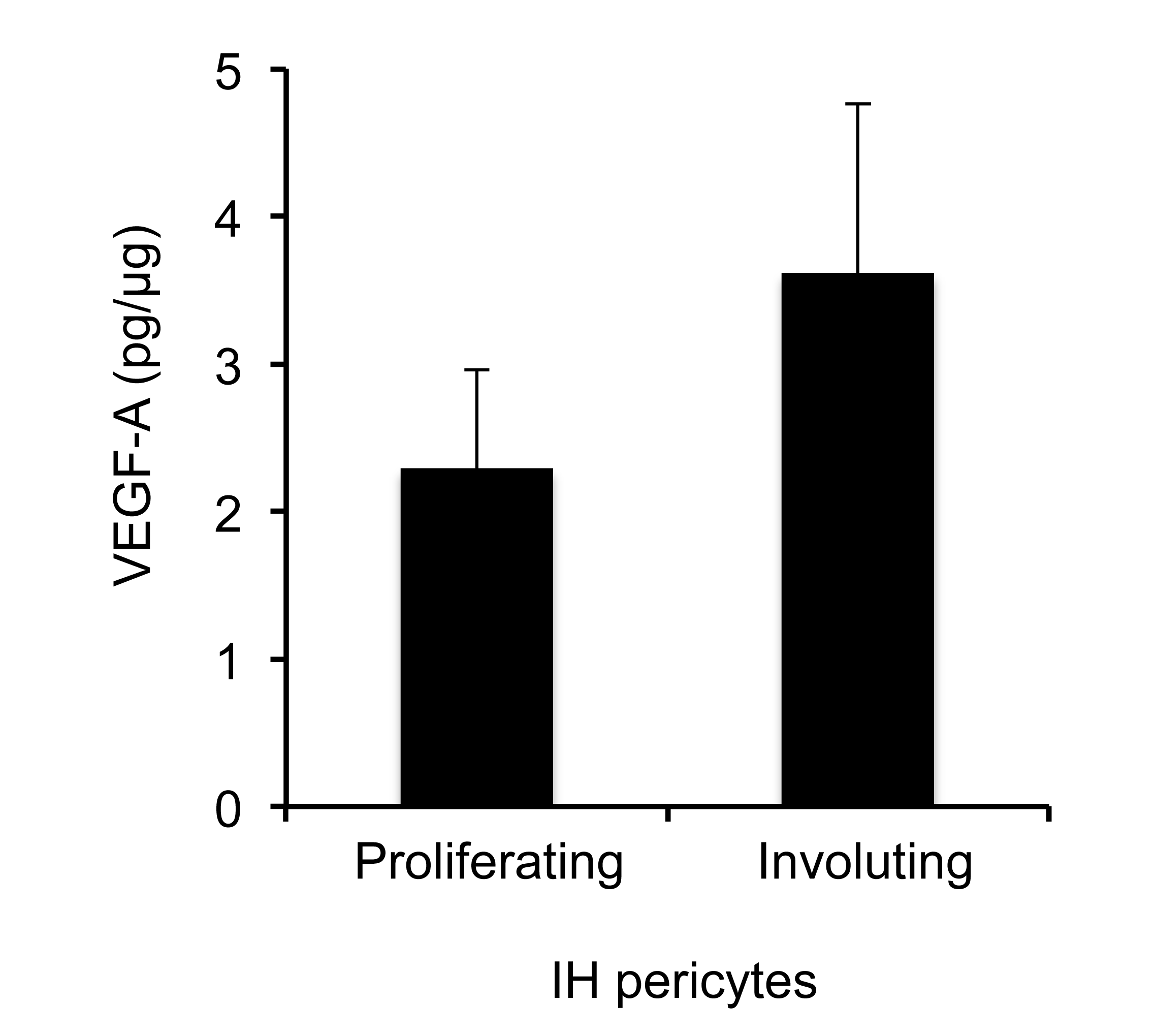

Supplement: Supplementary file 13 — Proliferating‐ and involuting‐phase IH pericytes express similar levels of total VEGF‐A. IH pericytes from proliferating and involuting phase express similar levels of total VEGF‐A. VEGF‐A165b was undetectable in these cells using ELISA [file PATH-239-139-s013.tif]

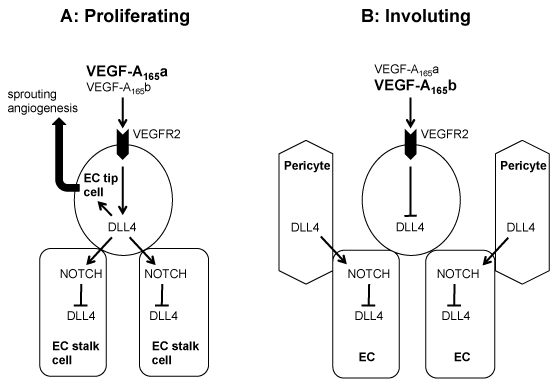

Supplement: Supplementary file 14 — Schematic representation of VEGF‐A and DLL4 interactions in IH. (A) High VEGF‐A165a activates VEGFR2 to mediate up‐regulation of DLL4 and subsequent establishment of the endothelial tip cells (EC tip cell); DLL4 from tip cells activates NOTCH in neighbouring cells to down‐regulate DLL4 and maintain a stalk phenotype (EC stalk cell); the tip cells guide the sprouting events in angiogenesis. (B) In the involuting phase, high VEGF‐A165b competes against VEGF‐A165a to prevent DLL4 up‐regulation and induction of tip cells does not occur; instead, DLL4 from neighbouring pericytes activates NOTCH in the endothelial cells to prevent up‐regulation of DLL4 [file PATH-239-139-s014.tif]
