# Supplementary material for: Altered ratios of pro‐ and anti‐angiogenic VEGF‐A variants and pericyte expression of DLL4 disrupt vascular maturation in infantile haemangioma
Source: J Pathol. 2016 May 13;239(2):139–51. doi: 10.1002/path.4715 (PMC4869683; doi:10.1002/path.4715)
Supplement: Supplementary file 15 — Primer sequences [file PATH-239-139-s015.docx]

**Table S1.** Primer sequences

| Target | Primer sequence (5′–3′) | Annealing temperature (°C) |
| --- | --- | --- |
| VEGF-A | GGAGGGCAGAATCATCACGAAG  CACACAGGATGGCTTGAAGATG | 58 |
| B_2_-microglobulin | TGCTATGTGTCTGGGTTTCATC  CCACTTAACTATCTTGGGCTGTG | 58 |
| VEGF-A_165_a and VEGF-A_165_b | TTGCTCAGAGCGGAGAAAGC  ATGGATCCGTATCAGTCTTTCCTGG | 55 |
